# Planning and optimising a digital intervention to protect older adults’ cognitive health

**DOI:** 10.1186/s40814-021-00884-2

**Published:** 2021-08-18

**Authors:** Rosie Essery, Sebastien Pollet, Kirsten A. Smith, Fiona Mowbray, Joanna Slodkowska-Barabasz, James Denison-Day, Victoria Hayter, Katherine Bradbury, Elisabeth Grey, Max J. Western, Alexander Milton, Cheryl Hunter, Anne E. Ferrey, Andre Matthias Müller, Beth Stuart, Nanette Mutrie, Simon Griffin, Tony Kendrick, Helen Brooker, Bernard Gudgin, Rosemary Phillips, Tom Stokes, John Niven, Paul Little, Lucy Yardley

**Affiliations:** 1 Centre for Clinical and Community Applications of Health Psychology, University of Southampton, Southampton, UK; 2 Department for Health, University of Bath, Bath, UK; 3 School of Psychological Science, University of Bristol, Bristol, UK; 4 University Hospitals Plymouth NHS Trust, Plymouth, UK; 5 Nuffield Department of Primary Care Health Sciences, University of Oxford, Oxford, UK; 6 Saw Swee Hock School of Public Health, National University of Singapore, Singapore, 117549 Singapore; 7 Centre for Sport & Exercise Sciences, University of Malaya, Kuala Lumpur, Malaysia; 8 Primary Care, Population Sciences and Medical Education, University of Southampton, Southampton, UK; 9 Physical Activity for Health Research Centre, University of Edinburgh, Edinburgh, UK; 10 Department of Public Health and Primary Care, University of Cambridge, Cambridge, UK; 11 University of Exeter Medical School, Exeter, UK; 12 Public and Patient Involvement (PPI) representative, Southampton, UK

**Keywords:** Cognitive-health, Behaviour-change, Physical activity, Dementia, Prevention, Digital-intervention

## Abstract

**Background:**

By 2050, worldwide dementia prevalence is expected to triple. Affordable, scalable interventions are required to support protective behaviours such as physical activity, cognitive training and healthy eating. This paper outlines the theory-, evidence- and person-based development of ‘Active Brains’: a multi-domain digital behaviour change intervention to reduce cognitive decline amongst older adults.

**Methods:**

During the initial planning phase, scoping reviews, consultation with PPI contributors and expert co-investigators and behavioural analysis collated and recorded evidence that was triangulated to inform provisional ‘guiding principles’ and an intervention logic model. The following optimisation phase involved qualitative think aloud and semi-structured interviews with 52 older adults with higher and lower cognitive performance scores. Data were analysed thematically and informed changes and additions to guiding principles, the behavioural analysis and the logic model which, in turn, informed changes to intervention content.

**Results:**

Scoping reviews and qualitative interviews suggested that the same intervention content may be suitable for individuals with higher and lower cognitive performance. Qualitative findings revealed that maintaining independence and enjoyment motivated engagement in intervention-targeted behaviours, whereas managing ill health was a potential barrier. Social support for engaging in such activities could provide motivation, but was not desirable for all. These findings informed development of intervention content and functionality that appeared highly acceptable amongst a sample of target users.

**Conclusions:**

A digitally delivered intervention with minimal support appears acceptable and potentially engaging to older adults with higher and lower levels of cognitive performance. As well as informing our own intervention development, insights obtained through this process may be useful for others working with, and developing interventions for, older adults and/or those with cognitive impairment.

**Supplementary Information:**

The online version contains supplementary material available at 10.1186/s40814-021-00884-2.

## Key messages regarding feasibility

- There is very limited evidence about whether digital delivery of multi-domain behaviour change interventions is feasible, engaging and acceptable in the context of protecting cognitive health. It is also unclear whether the same intervention content, structure and functionality is suitable for both those with and without existing cognitive impairment.
- This study demonstrated that a digital multi-domain behaviour change intervention can be acceptable and engaging amongst UK community-dwelling older adults. Iterative development of intervention content with users facilitated identification and resolution of potential barriers to acceptability and engagement. Importantly, there were no substantive differences between those with higher and lower cognitive performance scores regarding their intervention preferences, nor their ability and willingness to engage with it.
- These findings confirmed that the ‘Active Brains’ digital intervention content and activity recommendations should be suitable for older adults with and without existing cognitive impairment. Our subsequent feasibility and fully-powered trials will therefore test the same intervention amongst both of these groups.

## Background

Fifty-million people worldwide currently have dementia [1]. Cognitive impairment is even more common; mild cognitive impairment (MCI) and age-associated cognitive decline (AACD) are estimated to affect nearly 20% of adults aged 60 and over [2, 3]. Around 10% of MCI and AACD cases convert to dementia each year [4]. The annual global cost of dementia is nearly US$1 trillion with dementia prevalence expected to rise to 152 million by 2050 [5]. Dementia and cognitive decline place unsustainable demand on health and social care systems worldwide, and pose substantial threat to individuals’ independence and quality of life [6]. Prevention and management of dementia are public health priorities [7].

Increasing evidence suggests that health-related behaviours (e.g., physical activity and a Mediterranean-style diet) and cognitive training are protective of cognitive health [7–12]. Interventions targeting a single behaviour in individuals with and without existing cognitive impairment show some positive effects on cognitive outcomes [13–18]. However, findings are mixed and often inconclusive, prompting investigation of multi-domain intervention strategies [1, 7]. Complex behaviour change interventions are those that have several interacting components to try to achieve their aim [19]. Further to this, multi-domain interventions are those which target multiple risk factors and mechanisms for a particular condition [20], for example physical activity, cognitive training and healthy eating. Multi-domain interventions to prevent or reduce cognitive decline have shown mixed results [18, 21–23]. Despite positive effects of a face-to-face (group and individual) delivered programme addressing diet, physical activity, cognitive training and managing vascular risk [21], such interventions tend to be resource-intensive, prompting calls for scalable, cost-effective approaches [7, 18]. Understanding which intervention components are useful and how to improve cost-effectiveness is a key challenge [7]. Rapid development of advanced technologies and artificial intelligence will likely play a key role in future delivery of cognitive training in a way that is immersive and highly engaging for users, allowing adaptive personalisation and gamification [24–26]. In the meantime, comparatively ‘low-tech’ digital solutions may offer a cost-effective means of delivering cognitive training alongside other behaviour change intervention components.

Digital health-behaviour interventions have excellent potential to deliver content efficiently, effectively and accessibly at low cost [27]. There is early evidence that web-based multi-domain lifestyle programs may have potential for protecting cognitive health outcomes and dementia prevention [28] but much of this research is in very early stages. Furthermore, few of these studies test interventions that offer a combination of cognitive training and facilitation of health-related behaviour change. One pre-post design study showed promising effects of a digital intervention addressing behaviours including physical activity, diet, smoking, alcohol intake and sleep, but did not measure cognitive outcomes [29]. Furthermore, there is limited evidence about whether digital-delivery of interventions is feasible, engaging and acceptable in this context. Potential barriers to feasibility and acceptability relate to users’ cognitive capacity and digital literacy. It is important to explore whether individuals with cognitive decline have different preferences and requirements for intervention functionality. Furthermore, whilst older adults’ digital literacy is rapidly growing, there is still wide variation in ability and/or willingness to engage with digital health material [30, 31]. It is therefore important to explore whether digital content and functionality can be made accessible and engaging for these users, and how best to achieve this.

The aims of this paper are twofold: (1) to explore whether a digital approach appears to be a feasible, engaging and acceptable means of delivering a low-cost, multi-domain intervention to reduce cognitive decline amongst older adults; (2) to provide a clear account of how such an intervention was created through documenting its systematic development process. Clear reporting of the development of new interventions avoids ‘research waste’ and duplication of ineffective, unfeasible or unacceptable interventions [32]. This paper outlines the development of ‘Active Brains’: a digital intervention for 60–85 year-olds with and without existing cognitive impairment, aiming to reduce cognitive decline by addressing physical activity, cognitive training and healthy eating behaviours. We explain how the work conducted during this intervention development process allowed Active Brains to be shaped by target users’ expectations and preferences, whilst highlighting transferable insights and methods that could be applied across numerous behaviour change contexts.

## Methods

### Structure of the development process

**Fig. 1:**
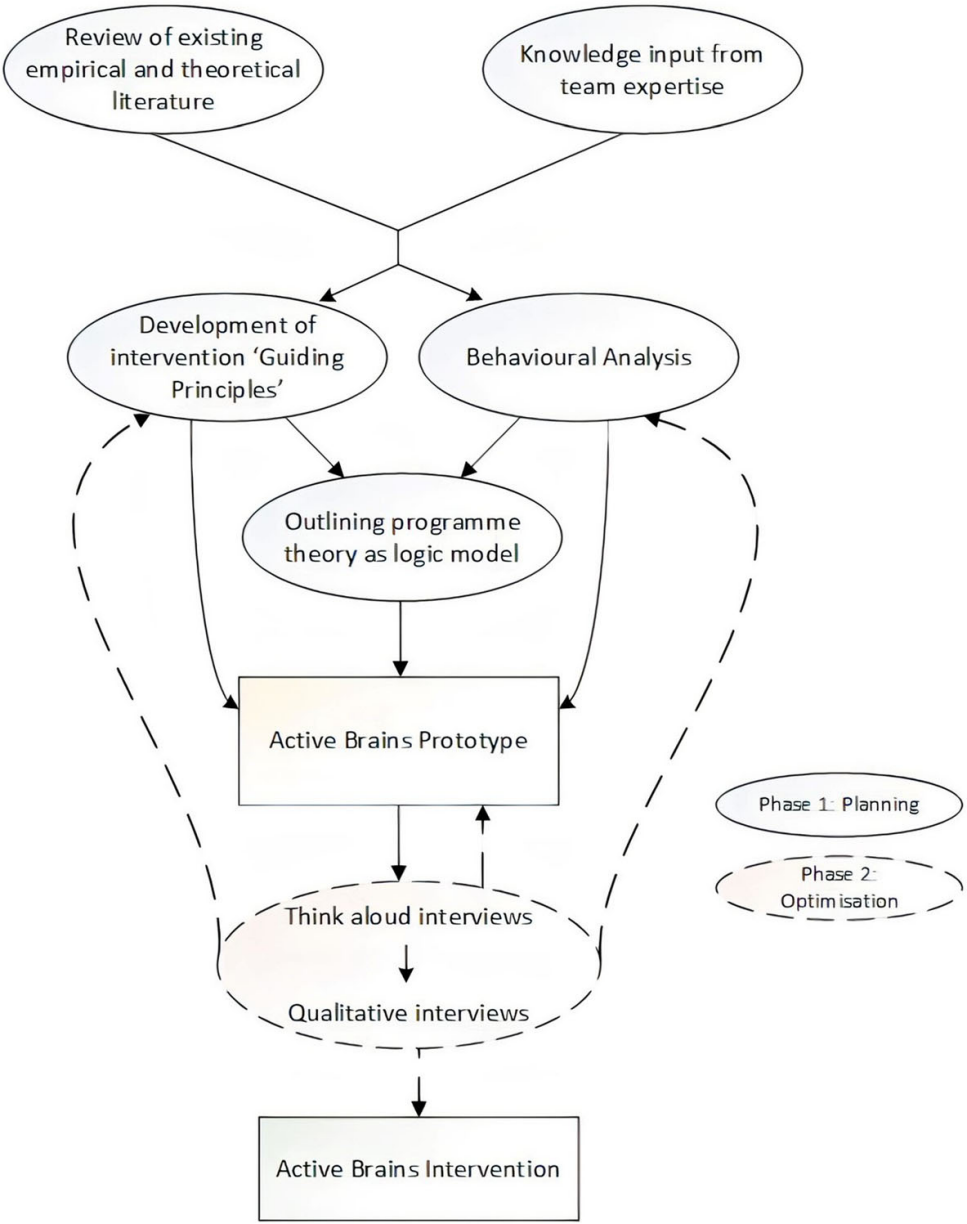
Planning and optimisation processes involved in the development of Active Brains

Active Brains was developed according to a theory-, evidence- and person-based approach to intervention development [19, 33, 34]. The development process was implemented, and is outlined below, in two phases: phase 1: planning and phase 2: optimisation. The ‘Planning’ phase presents the theory-, evidence- and person-based ‘Guiding Principles’ [34] and logic model that underpin intervention content and functionality. The ‘Optimisation’ phase presents our qualitative findings about older adults’ perceptions of cognitive health and associated protective health behaviours, as well as their feedback on all aspects of intervention content throughout development. Although described separately, in practice, these phases occurred as an iterative cycle (Fig. 1). Development focused on the physical activity and cognitive training intervention elements as the team had previously developed a healthy eating module that could be adapted for use in this context [35]. Additional Table 1 summarises how each element of the Active Brains development process addresses recommended actions for intervention development [32].

Monthly development meetings were held with a multi-disciplinary team of coinvestigators including Patient and Public Involvement (PPI) representatives. PPI members played a significant role in ensuring that study and intervention materials were accessible, engaging and persuasive prior to being shared with participants. The wider management team met quarterly and included GPs, specialist clinicians, health psychologists, dementia charity partners and academics with expertise in physical activity, cognitive health and nutrition. Draft intervention materials were frequently shared for comment and iteration.

The following sections outline the methods employed in: phase 1, planning the intervention’s theoretical framework; and phase 2, the empirical qualitative work conducted to optimise Active Brains. Respective findings/outcomes from each of these processes are reported in the results section.

### Phase 1: planning Active Brains

The planning phase aimed to build the appropriate theory-, evidence- and person-based framework to underpin the Active Brains intervention. This involved reviewing relevant literature, seeking input from PPI contributors and co-investigators with relevant expertise, developing guiding principles, conducting a behavioural analysis and constructing a logic model.

#### Phase 1, part 1: reviewing relevant literature

We conducted rapid scoping reviews of (1) physical activity and/or sedentary behaviour interventions, and (2) cognitive training interventions, for older adults with and without cognitive impairment. We aimed to gather evidence about: promising intervention features; relevant contextual factors; and important influences on targeted behaviours. Searches were conducted in Web of Science, March–June 2017 (Additional Table 2). Additional literature was identified through reference-list searching and consultation within our team. Quantitative and qualitative papers were included. Initial searches returned over 9000 matches about cognitive training interventions including a significant number of existing systematic reviews in this field. Therefore, we focused only on systematic reviews (*n* = 14) given that these had collated, and quality assessed, a wealth of evidence about existing cognitive training interventions. Data were extracted about research design, sample size and characteristics, and findings.

#### Phase 1, part 2: development of guiding principles

Guiding principles aim to maximise the acceptability of an intervention amongst target users and, therefore, to enhance engagement and effectiveness. Each guiding principle comprises (1) a design objective outlining a user/context-specific behavioural need, issue or challenge; and (2) intervention features that address the design objective [34]. To draft provisional guiding principles, we drew on our understanding of target users obtained from the scoping reviews and from our research team, including PPI members. These guiding principles were iteratively developed as new data emerged, e.g. from the behavioural analysis and qualitative interviews.

#### Phase 1, part 3: developing Active Brains programme theory

A programme theory explicitly describes how an intervention is expected to achieve its intended outcomes, and the anticipated mechanisms through which this occurs [36]. The behavioural analysis and logic model contributed to this process.

##### Phase 1, part 3a: behavioural analysis

The behavioural analysis aimed to identify behaviours to be targeted by Active Brains and their potential barriers and facilitators. We recorded relevant evidence from scoping reviews, PPI input, team expertise and later qualitative interviews into a table, and mapped the identified behaviours and potential barriers and facilitators onto constructs from the behaviour change wheel (BCW) [37] and theoretical domains framework (TDF) [38] in order to describe target domains for our intervention in theoretical terms. Given that so many different behaviour change theories have overlapping constructs, it can be difficult to identify any one individual theory to apply [39, 40]. Accordingly, we drew on these theoretical frameworks derived from multiple theories that encapsulate a wide range of theoretical constructs. This allowed clear description of the intervention processes and components, including behavioural domains to be targeted, intervention functions to address barriers and facilitators and behaviour change techniques BCTs [41]; to deliver these functions. In addition, this process facilitated identification of other potentially useful domains to target, and additional intervention functions and BCTs to employ that may maximise the intervention’s possible effects on its target behaviours [37].

##### Phase 1, part 3b: the Active Brains intervention logic model

In line with Medical Research Council (MRC) guidance [42], we constructed a logic model to diagrammatically represent the expected mechanisms of action of Active Brains. This drew on the scoping reviews, team expertise, guiding principles and behavioural analysis.

### Phase 2: optimising Active Brains

The optimisation phase aimed to seek feedback on draft intervention material, and to explore the acceptability and feasibility of the digital content and functionality amongst older adults with higher and lower levels of cognitive performance. Due to the vast quantity of relevant literature available to inform initial content development, as well as substantial expertise and experience to draw on from our project team and PPI, primary qualitative research was delayed in favour of planning and drafting initial content [43]. This was deemed appropriate given the depth of understanding we felt we could obtain from the existing literature and both PPI and expert co-investigator knowledge to inform our guiding principles. This allowed us to seek feedback on initial drafts of our intervention content sooner, and meant we could, and did, still explore target users’ experiences to inform changes required. Our iterative development process meant that our target users’ views still closely informed content development from an early stage. This included returning to elements of the planning phase, such as guiding principles and behavioural analysis, to revise these as and when our primary qualitative work indicated that we may need to change or add to our previous assumptions.

#### Phase 2, part 1: think-aloud interviews

##### Participants

Forty-one adults (22 female, mean age = 70.5 years, range 61–80) were recruited from GP practices across the South of England and from Join Dementia Research (JDR; an online database for matching UK community-dwelling individuals to relevant studies) to take part in think-aloud interviews. We employed purposive sampling whereby we attempted to obtain maximum variation in terms of gender, age, education level, socio-economic status and cognitive performance score. Participants were excluded if they were already reasonably physically active (score > 30 on Godin Leisure Time Exercise Questionnaire [44]), had diagnosed dementia, a severe uncontrolled mental health condition, or terminal illness. As part of the screening process, participants completed a brief cognitive assessment (online Baddeley verbal reasoning task [45]), which determined whether they were identified as a participant with ‘lower cognitive performance’ or ‘higher cognitive performance’. Lower cognitive performance was defined as a score falling more than one standard deviation below the ‘normative score’ on the Baddeley verbal reasoning task (i.e. in line with definitions of AACD [46]) as determined by the PROTECT cohort database; scores from a large (*n* > 15,000) pre-existing cohort of older adults [47]. Although this single test was not indicative of cognitive impairment, this categorisation enabled us to sample views from those with higher (*n* = 20) and lower levels (*n* = 21) of cognitive performance.

##### Procedure

Each participant took part in one think-aloud interview in which they worked through the prototype Active Brains intervention with an interviewer. The participant was encouraged to vocalise all immediate thoughts and feelings towards the content. This allowed insight into target users’ immediate reactions to elements of the intervention. As it was unfortunately not possible for participants to access the brain training games, we provided screenshots to show the types of task this involved. The Active Brains intervention provides access to brain training games via the PROTECT study; these existing games have been extensively used in ongoing cohort studies, and study investigators report them being well liked and engaged with [11, 47]. Following the think-aloud interview, there were semi-structured interview questions about participants’ general views of the intervention: what they liked/disliked, found helpful/difficult, would like to change, etc. All interviews were audio-recorded and transcribed verbatim.

##### Analysis

Data were analysed to understand user views on the intervention content and inform potential changes. We collated all positive and negative comments pertaining to specific intervention elements into a ‘table of changes’ (Additional Table 3). After discussing the frequency and significance of positive and negative comments , we coded the importance of possible changes by deciding whether any amendment was likely to enhance the persuasiveness, acceptability and likelihood of changing behaviour [48]. For example, we considered whether multiple people provided the same feedback; if the potential change aligned with our guiding principles and/or expert opinion; and whether theory and/or evidence suggested the change would make the desired behaviour more likely. We prioritised changes by their relevance to behaviour change or ability to prevent disengagement. If changes were low-priority, they were implemented only if relatively quick and easy. Interviews continued alongside this analysis to allow iterative modification of content prior to the next batch of interviews. Once it seemed that no further important changes were required, we considered that data saturation had been reached [48].

#### Phase 2, part 2: retrospective qualitative feasibility study

##### Participants

This second element of the qualitative work commenced once the majority of prioritised modifications to the prototype had been made. Eligibility, sampling and recruitment procedures were the same as in the think-aloud interviews. Eighteen older adults (12 female, mean age = 69.1 years, range 62–76) took part, seven of whom had participated in the think-aloud interviews. The eighteen participants were classified evenly across the lower and higher cognitive performance groups (*n* = 9 in each group).

##### Procedure

Participants were invited to use Active Brains for three weeks (timings were ‘sped-up’ to allow access to all sections) and were given a diary to keep notes about their experiences. Participants took part in one semi-structured interview each during this time. The interview asked participants about their experiences of engaging with the intervention and any relevant activities they tried. They were prompted to discuss certain features or elements that they particularly liked and/or found helpful or disliked and/or found difficult. Towards the end, there were questions about participants’ perceptions and understandings of cognitive health, and their views on social support for engaging in new activities. All interviews were audio-recorded and transcribed verbatim.

##### Analysis

All data were tabulated and analysed as described in the think-aloud study (phase 2, part 2a). In addition, inductive thematic analysis [49] was conducted on the data from the second part of the interview examining perceptions and understandings of cognitive health and social support (phase 2, part 2b).

## Results

### Phase 1: planning Active Brains

#### Phase 1, part 1: reviewing relevant literature

Given the wealth of existing reviews on the topics of interest, the findings were not formally synthesised for write-up. However, key findings pertinent to our research aims are summarised in Table 1, which also illustrates how they informed intervention guiding principles. Key findings included there being no substantial evidence that the intervention’s physical activity recommendations should differ for older adults with MCI/AACD compared to a general older-adult population. The cognitive training systematic review evidence suggested training multiple cognitive-domains to be the optimum choice for both cognitively-healthy older adults and those with cognitive impairment e.g. [50, 51]. Regarding physical activity interventions, those with and without cognitive impairment shared similar attitudes towards physical activity, and recognised similar barriers (e.g. remembering, social isolation), facilitators (e.g. accessibility of activity options, simple activities) and preferred activities (e.g. walking) [52, 53]. There was only a small amount of evidence about intervention features that may be acceptable and engaging for both groups. Acceptable intervention features amongst those with cognitive impairment often overlapped with those frequently used in interventions for older adults in general e.g. planning features [54];. Otherwise, there was little evidence about whether engagement with intervention features was likely to differ between groups, so we aimed to explore this within our primary qualitative work.

**Table 1:** The Active Brains guiding principles

Key findings from literature	Key design objective	Intervention feature(s)
Older adults with cognitive impairment tend to experience difficulties in the domains of memory, language, thinking and judgement. Difficulties not so extensive that the individual requires assistance with activities of independent daily living [55, 56] Older adults with cognitive impairments that may affect Internet use are still actively engaging with technology [57] Good evidence of effectiveness and/or acceptability of various features/characteristics of interventions: - Simple goal setting and action planning with clear explanation of benefits/ importance [58] - Reinforcement/encouragement for achievements [59] - Self-monitoring of physical activity behaviours, e.g. using a pedometer [58, 60] - Social support in the form of activity suggestions to be done with others/ local group recommendations [59, 60]; social element of cognitive training may also be beneficial [61] - Promotion of autonomy [59] Strength and balance exercises can be built into daily routines and activities [62]	Minimising cognitive load and dependence on technology	• Clear and simple layout, language and navigation procedures • Support provided for cognitive self-regulation (e.g. planning, reminders, prompts for periodic short-term and long-term self-monitoring) • Utilising non-cognitive/non-digital means of sustaining behaviour (habit formation, environmental restructuring) • Options to print/ save key reference documents/ instructions wherever possible. • Link to existing non-digital sources of advice/ support where appropriate, including peer/ family support if possible
Individuals more motivated to participate in, and have better recognition memory for, physical activity programmes paired with positively framed messages than in those with negatively framed ones [63] Loss of independence perceived as key threat of cognitive decline [64] Older adults with cognitive impairments very interested in programs offering computer exercises to improve cognition as well as web-based interventions for a range of health concerns and lifestyle factors, including physical activity, diet and nutrition, social engagement [57] Enjoyment of activities is important [52, 53, 65] Different/new activities such as strength, flexibility and balance exercise may be beneficial for long-term engagement [66] Need for evidence-based, credible communication of link between increasing physical activity and cognitive health [64]	Positive framing and promoting immediate-term quality of life benefits	• Framing activities in terms of benefits for: maintaining independence, enjoyment, strength, balance, pain (especially musculoskeletal), mood, general quality of life. • Referring to benefits for Brain Health rather than reduction in dementia risk
Tailoring for different levels of mobility, having optional exercises important/ preferred [58, 59, 67, 68] Need for activities to be simple and safe highly prioritised [52]	Catering for highly heterogeneous population (capabilities and preferences)	• Tailoring of content to offer options for levels/ types of activities, with steer towards those with best evidence and most likely to be beneficial for user (based on baseline-assessed need and capability, e.g. activity levels, perceptions of current strength and balance skills) • Provision of carefully graded activities with very gradual increases from low activity baseline and help with concerns and barriers for those lacking confidence or capability

#### Phase 1, part 2: development of guiding principles

The finalised Active Brains guiding principles (Table 1) were minimising cognitive load and dependence on technology; positive framing and promoting immediate-term quality of life benefits; and catering for heterogeneous preferences and capabilities. These guiding principles underpinned and informed the development of all intervention materials both in terms of the content, and also the presentation style, format and functionality.

#### Phase 1, part 3: developing Active Brains programme theory

##### Phase 1, part 3a: behavioural analysis

The full behavioural analysis is presented in Additional Table 4. Active Brains targeted nine behaviours: initial engagement with the online intervention; increasing physical activity; reducing sedentary behaviour; uptake of strength and balance activities; uptake of brain training; healthy changes to eating behaviours; reviewing behaviours and revising goals; integration of recommended activities into daily routines; and maintaining engagement with the online intervention. These behaviours were further broken down into 19 sub-behaviours required to enact each behaviour. Mapping these behaviours, their determinants and intervention features onto the BCW and TDF illustrates that Active Brains employs 36 BCTs to deliver seven intervention functions (modelling, education, persuasion, training, enablement, environmental restructuring, incentivisation) to target thirteen behavioural domains (intentions, optimism, emotion, knowledge, skills, beliefs about consequences, beliefs about capabilities, goals, social influences, environmental context and resources, reinforcement, memory, attention and decision processes and behavioural regulation). This analysis provided an in-depth understanding of the behaviours for Active Brains to target and the mechanisms through which it is anticipated that these could be changed. These understandings informed the development of the intervention’s logic model.

##### Phase 1 part 3b: the Active Brains intervention logic model

**Fig. 2:**
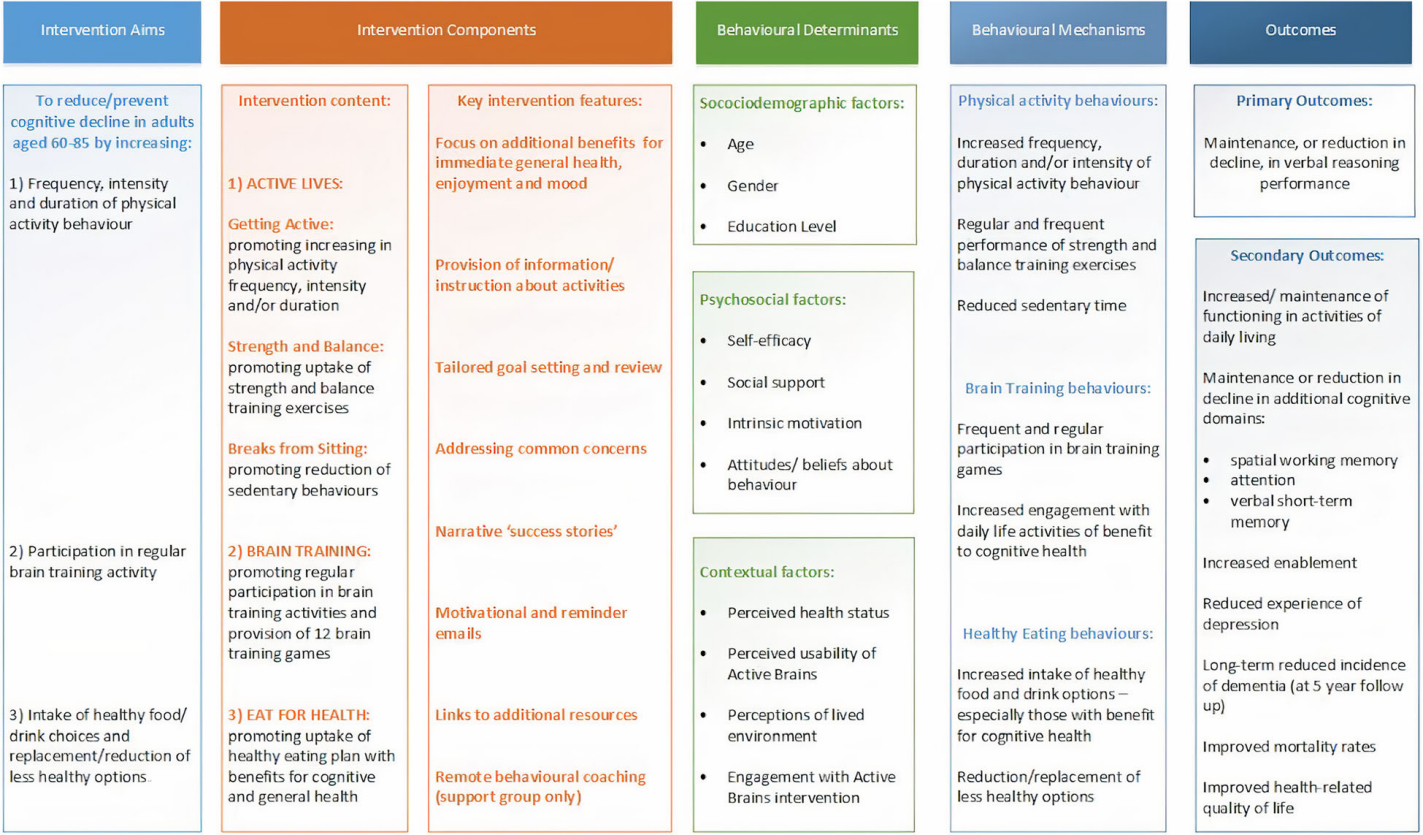
Active Brains summary logic model

A summary version of the Active Brains logic model is shown in Fig. 2. Additional Figure 1 shows the full version with intervention processes mapped on to BCW, TDF and BCTs. The culmination of the planning phase in preliminary guiding principles and a logic model provided the underpinning framework for Active Brains. The Active Brains digital intervention comprises three online modules that become available sequentially: ‘Active Lives’ (physical activity) is available immediately; ‘Brain Training’ (cognitive training) is available after 4 weeks; and ‘Eat for Health’ (healthy eating) is available after 8 weeks. ‘Active Lives’ is further divided into three sub-modules: ‘Getting Active’, ‘Strength and Balance’ and ‘Breaks from Sitting’ with recommendations about which to start with tailored to users’ baseline activity and capability. Within each module, users can access information addressing common concerns, instruction about recommended activities, goal setting and review for chosen activities and tailored motivational feedback on progress. Reminder emails are sent to motivate users to continue with their activities and to encourage them to revisit online content. Additional support from a central facilitator (for one arm of Active Brains trial) comprises up to three 10-min phone calls at 2-week intervals, plus additional email support if required. This can be used to discuss behavioural changes participants are attempting, and to support them with use of the online intervention content. The facilitator employs the CARE (Congratulate, Ask, Reassure, Encourage) approach to provide support in a broadly standardised format [69]. After 7 months, the Active Brains ‘booster section’ allows users access to additional resources for embedding recommended activities into daily life. It also introduces the brain training ‘boosters’ to maintain the benefits of the initial intensive training period.

### Phase 2: optimising Active Brains

The findings of the qualitative work are described below. These fed back into ongoing iteration of the guiding principles, and behavioural analysis and also informed required intervention changes.

#### Phase 2, part 1: think-aloud interviews

Feedback on the Active Brains prototype was encouraging with largely positive feedback from participants’ indicating that they found the content easy to understand, persuasive and interesting. Users were particularly positive about what they considered to be more novel activities including strength and balance training, and brain training games.

I thought it was actually really helpful, and I thought it offered a really wide range of ways for people, starting from different levels of activity, to think about doing more. I also thought the parts that are the little sections that said things like 'I'm concerned about overdoing it', you know the sections about people's concerns? I thought that the content of all of those parts addressed the issues really clearly. (J0105, female, 65, higher cognitive performance)

I thought it was very good actually. I thought it was excellent in fact. If only for the fact that it did, it related to me one hundred percent. It was completely informative and helpful, you know, giving… giving me the impetus to move on. (P0122, male, 73, lower cognitive performance)

Less positive feedback included sections where users found navigation confusing, a lack of specificity surrounding physical activity goals, and a desire to address health-related concerns earlier. We analysed feedback for differences between those with lower and higher cognitive performance scores to determine whether different intervention features or characteristics may be more engaging or desirable for those with lower cognitive performance. There was no evidence of any substantive differences. Table 2 summarises key feedback that required addressing and the resulting changes implemented.

**Table 2:** Summary of feedback and amendments resulting from think aloud interviews

Active Brains section	Summary of issue identified	Example	Change implemented
**Introductory content**	Worry about continuing before being reassured that it would be safe to try new activities with various health conditions/ other concerns	“I would have it earlier, yeah, because the question in itself is too sort of set, you know, it, it’s about increasing activity gradually rather than overdoing it. And I think something about that needs to go before. (P0136)	Although each subsection contained a section addressing concerns, it was deemed important to bring shared concerns forward to the introductory material so people felt happy to proceed with the intervention content
	Some pages (and this applied throughout) perceived to be a bit cluttered/ busy with too much text which some found off-putting	“I immediately look at this page and find it untidy and as a, not a struggle, but as a barrier there to reading it clearly and understanding it. I’m struggling to find what to click to go to next.” (P0245)	Focusing on identified problematic pages, we edited text to a minimum. Wherever possible text was bullet-pointed and only key messages retained. If important to keep all text on a page, this was split over multiple pages where appropriate.
**Getting Active**	Uncertainty about goal setting: some seemed unsure about exactly what they had set themselves goals to do even when goal setting process complete	“… it’s good to have goals, but I think the goals need to be specific. If you're asking people to achieve a goal that's very vague, I don't think they're enthusiastic and I think they give up and they probably give up the whole thing.” (P0111)	After revisiting the activity suggestions made in this section, it was considered that this uncertainty was likely to be arising from the fact that the activity suggestions and plans that people could select were a little too broad—these were amended to more specific options for people to choose from
	Many mistaking coloured, bolded text (to emphasise key messages in text) for hyperlinks and expected links to additional content	“…anything else that's in blue, you think you can click on it”(P0102)	We removed the colouring of these parts of text, but retained the bolding for emphasis. The minimisation of text to key messages only also addressed this issue.
**Strength and Balance**	Concern that information provided about the principles of how strength and balance exercises worked (including information about specific movements such as shifting weight to one side) was potentially risky if attempted by those who were less mobile.	“…if you have somebody with poor balance, it’s just trying to ensure, how do you ensure that someone who shouldn’t really be standing on one leg doesn’t stand on one leg, despite what you’ve said about being safe.” (P0106)	The text in this section was reframed to describe the underlying principles of the exercises without reference to specific examples that may be dangerous if attempted by someone with poorer mobility/balance. Instead it now talks about how the suggested activities allow practice of movements to expand individuals ‘comfort zone’ in terms of movements they can make.
	Disagreement with advice that if users are unsure about whether suggested activities are suitable for them, then to consult with their GP – users not comfortable with the idea of taking up GP time with these types of queries.	“It’s… I always find this information about checking with your doctor before you start interesting, because I very… well, I say that. I very seldom make plans to go and visit the doctor. And I certainly wouldn’t regarding this, I think.” (P0105)	Revised to reassure users that the activities recommended were nothing outside of routine movements made in day-to-day life and that they were likely the best judge of whether they could safely/comfortably do these Also encouraged to discuss with those who knew them well, and only seek advice from GP for serious concerns.
**Breaks from Sitting**	Some activity plans about how to implement recommendations deemed unrealistic or ‘silly’—e.g. purposely leaving objects upstairs that you know you will need downstairs to increase steps around the house	“Leave your bedtime book on the kitchen table. I don't get that one. How does that work? You're just gonna pick it up when you're downstairs and carry it to the bedroom and then bring it back down…” (P0101)	Such examples were removed or replaced. New suggestions focused on ways people could: add some movement in to times they might be completely sedentary (e.g. leg raises whilst watching TV); add more movement into standing or mostly sedentary times (e.g. marching on the spot whilst brushing teeth)
	Some activities to identify common times/places for sitting, were not considered either: a) a suitable target for change (i.e. at doctors surgery), or b) an activity that they would actually do sitting down (e.g. brushing teeth)	“Yeah, I think if you stood up when you were waiting to see a doctor or nurse… I think you'd… people don't stand up, do they?” (J0112)	These suggestions were removed
**Brain Training**	The wording of some True/False quiz questions was considered confusing, e.g. one stated that the purpose of brain training games was to keep improving your score. If/when people answered ‘True’, they were surprised when their answer wasn’t considered correct	“Yeah, well that’s, that automatically to me should be true but it’s, you explaining it, but it’s not clear. The point of brain training is for you to get better, any training is to get better, but it said it’s false.” (P0104)	To ensure participants remained engaged with these quiz questions and did not take away the wrong message, wording/ feedback was revisited and amended where necessary. In this example, the feedback was clarified to state that whilst a good aim to try and improve scores, the important factor is continuing to practice these games whether or not your score improves
	Given different structure of the Brain Training section compared to other sections, a page preceding the Brain Training menu explained how to use menu page, but this created confusion	“It's a bit confusing, this one. I don't know quite why. This bit I think might be more beneficial on the next page?” (J0112)	This page was removed and the navigation around the Brain Training menu pages was revised to ensure that it was clear how users could access each element of the Brain Training module—most importantly the link to the Brain Training games.

#### Phase 2, part 2: retrospective qualitative feasibility study

##### Phase 2, part 2a: table of changes analysis

Collating feedback from this later round of interviews into a second table of changes confirmed that the amendments based on the initial think-aloud interviews were well received, with the original issues no longer being raised. Participants’ accounts of their experiences also revealed examples of ways in which they had engaged in the activities recommended by the intervention and confirmed they were happy with the digital delivery format.

It did make me think about it in general, and reminded me that I'm not doing so much aerobic activity, and I'm not really measuring my activity. So I decided I would - there are about four flights of stairs when I go to work, and I always used to walk up them, and now I've got a bit lazy about it, so I decided I was going to go back to that, and also use an app to measure how much I walk, because I've got a dog and I walk a lot every day. (P0129, female, 67, lower cognitive performance)

I found the explanations on the type of foods you should eat to help your brain. I found all those very interesting. I don't think my diet is that bad, but it's nice to know that I have been eating the right things and things that I can add too, to what I'm doing. I like the recipes. I'm looking at the recipes, I did print those out. (P0229, female, 68, higher cognitive performance)

But I mean somebody who hadn’t got any [IT] skills and were just having to read and have just got a next or a back button, it is very easy to use. You don’t really need to do much, as long as they know where the click on and off, and move on. It’s like turning a page of the book, isn’t it? It’s as simple as that, isn’t it? Yes. (P0104, female, 75, lower cognitive performance)

In general, there were a smaller number of negative comments about the intervention content, but a few remaining points were identified and addressed (Table 3). There were no substantial differences in the views expressed by individuals with higher and lower levels of cognitive performance.

**Table 3:** Summary of feedback and amendments resulting from retrospective interviews

Summary of issue identified	Example	Change implemented
Some voiced opinion that they felt physical activity content wasn’t relevant to them as they perceived themselves to already be physically active (despite not meeting exclusion criteria for existing high levels of physical activity)	“For me, it was the actual activities, the actual physical bits, weren't terribly challenging.” (P0146)	Additional messages added in to introductory and early physical activity content to emphasise that even those who are already active can use content to help them increase activity, and to stress importance of continuing with/ increasing activities they already do and enjoy. Additional signposting to aspects of content where users have more free choice about the level of challenge of their activities—e.g. writing their own goals, resources for finding activity classes/groups in their local area, and setting their own strength and conditioning exercise plan,
A few suggestions that it would be useful to have more explicit suggestions about ways to stay motivated with making behavioural changes—particularly about how to use social support to do so	“The one thing I think you could do a bit on is finding the incentive to do all these things, so that we've got to do them to keep going, but you tend to put them off because you're doing other things at the time.” (P0265)	Extra pages added to give examples of motivational strategies, including ways to involve others (e.g. weekly step-count competition with friends/family) and activities to boost motivation (e.g. ‘Reasons to be Active’ card).
Some perceived healthy eating content to be largely in line with what they were already doing. Furthermore, expert advice from within the team recommended placing greater emphasis on the ‘foods for brain health’ as novel/interesting element.	“I read it through with interest and I thought: Oh, well, I do that; I eat that. I agree with all of that and that's what I do; but there was nothing in there that I felt that I didn't already do.” (P0250)	Restructure of the healthy eating content so that the initial information and goal setting centres around specific foods beneficial for cognitive health, with additional more general healthy eating advice presented after this.

##### Phase 2, part 2b: inductive thematic analysis

The inductive thematic analysis generated three overarching themes, comprising several subthemes. These were (1)‘knowledge and understanding of brain health’, including subthemes ‘the meaning of brain health’, ‘perceived availability of information about brain health’ and ‘knowledge of determinants of brain health’; (2) ‘motivators and barriers’, including the subthemes ‘motivations for achieving/maintaining good brain health’, ‘motivators for engaging in helpful behaviours’ and ‘barriers to engaging in helpful behaviours’; and finally (3) ‘the role of social support’ including subthemes ‘desirability of social support’ and ‘motivational mechanisms of social support’. Key findings from each theme are briefly summarised with illustrative quotes from the data. These findings helped to further refine the intervention guiding principles and behavioural analysis.

##### Knowledge and understanding of brain health

This theme suggests that, for older adults, ‘good brain health’ is largely about maintaining independence and remaining able to do the activities one wishes to do. More than half of participants also discussed retention of specific cognitive skills such as good memory and decision-making.

If you've got good brain health, then you can carry on with your daily life: cooking, managing your finances, managing your social life—you know, day-to-day things, really. (P0229, female, 68, higher cognitive performance)

A large proportion of individuals felt that, whilst information about cognitive health and how to protect it is available, it often requires one to actively look for it. Many also mentioned the availability of information about body health, but not necessarily about brain health.

So you do need to know about it. But you have to make the effort to either read a newspaper or look at the news, or get your brain active yourself. (P0265, female, 69, lower cognitive performance)

Despite this, nearly three-quarters of participants named typically promoted strategies for maintaining cognitive health, such as brain training activities and puzzles. Half of participants also acknowledged the role of health-related behaviours, such as physical activity, in maintaining cognitive health.

##### Motivators and barriers

Two different types of motivation were identified within participants’ accounts. The first were motivations to maintain good brain health in order to avoid cognitive decline and its anticipated negative consequences, such as loss of independence, poor quality of life and interference with relationships. This was often accompanied by accounts of friends or family with dementia and their strong wish to avoid this.

It’s a tremendous thing, for me anyway, because I've seen other people go through it. I don't want to, […] It is frustrating for other people as well as for yourself. I think it's important not just for you, but it's also important for the rest of the family, and to be able to pass the memories on as well. (P0225, female, 65, lower cognitive performance)

The second type of motivation related to factors that encouraged individuals to engage in behaviours important for maintaining cognitive health. The overwhelming sentiment was that enjoyment is the main motivator. Even when individuals acknowledged that behaviours were beneficial for brain health, this seemed an ‘added bonus’ rather than the primary motivator.

…yeah, you know, I do a lot of things like maths games. And crosswords and stuff like that every day, so I don’t know if that actually helps but I just find them interesting. (P0138, male, 70, higher cognitive performance)

Barriers to engaging in activities to support cognitive health were not discussed extensively, but the most common difficulty mentioned was managing other health conditions.

I'm quite hampered with physical activity because I've got arthritis and am registered disabled so, to be honest, physical activity is so difficult for me. That's where these exercises come in, really and it's mostly what I can do. (P0261, male, 62, higher cognitive performance)

##### The role of social support

Participants who discussed involving others in healthy lifestyle activities mentioned several mechanisms through which this provided motivation for beginning and maintaining activities. This included creation of action plans with others, being accountable to others and sharing encouragement and new ideas.

I think, if you're going swimming or something once a week, it's nice if someone says, 'Are you ready to go?' 'Shall we go today?' rather than you think: Oh, do I really want to go today? If there's two of you or three of you wanting to go, you encourage each other. (P0229, female, 68, higher cognitive performance)

However, it was widely acknowledged that individuals’ preferences and circumstances determine whether involvement of others is possible, or even desirable. More than half of participants expressed that they would be happy (or sometimes prefer) to do such activities alone.

I’m quite happy with my own company. I mean, I enjoy doing things with other people, and I go to yoga and I get on with everybody there, and I've got quite a few friends that go, but I would go whether they went or not. (P0129, female, 67, lower cognitive performance).

## Discussion

This paper presents a theory-, evidence- and person-based approach to intervention development that could be applied across numerous behaviour change contexts. We have provided a systematic account of how and why the intervention took its current form, and how it is expected to work. In doing so, we have provided important provisional insights into the likely acceptability of a digital multi-domain intervention to reduce cognitive decline amongst older adults with a range of cognitive performance abilities. This forms the basis for further work which will next explore the feasibility [70] of this intervention amongst a much larger sample. Furthermore, this work illustrates the application of a systematic process for intervention development that can be applied widely.

Key outcomes from each component of this study collectively provide preliminary evidence that a digital multi-domain behaviour change intervention can be acceptable and engaging amongst UK community-dwelling older adults. The study’s embedded qualitative work with intervention target users also allowed iterative optimisation of our draft content to ensure barriers to acceptability and engagement were identified and addressed. Furthermore, our findings about older adults’ perceptions of cognitive health facilitated understandings of characteristics and features of intervention content that would be important for acceptability and engagement. These findings generally align with the existing literature: for example, maintaining independence [64] and enjoyment of activities [65] seem key motivators amongst this group. We also found that an awareness of, and desire to avoid, the consequences of dementia seemed to motivate cognition-protective behaviours [71], whilst other health conditions arose as a possible barrier. Additionally, our findings extend understandings about the value of social support. Whilst they concur that, for many, social support is an important motivator of behaviours such as physical activity [60], they reveal that for others it is not considered necessary or desirable. This has important implications for offering social support within interventions, i.e. it should be available but not a compulsory element. Furthermore, as social support didn’t appear to be a primary motivation for engagement with the intervention, this lends additional support to the potential feasibility and acceptability of a digital-delivery format.

Our findings also reinforce and extend existing literature about the suitability of intervention content for individuals with varying levels of cognitive performance. Importantly, we found no evidence of qualitative differences between those with higher and lower cognitive performance scores regarding their preferences or requirements for the Active Brains intervention, or their ability and willingness to engage with the digital intervention. This finding reinforced our judgement (informed by the initial literature review) that Active Brains’ content and delivery-format may also be accessible and engaging for people with lower cognitive performance scores and did not need to be tailored according to cognitive status. This supports previous findings [50, 51], suggesting that the same activity recommendations might be suitable for those with and without cognitive impairment, given that they appear to share similar motivations, barriers, and attitudes. The findings regarding the acceptability of the digital delivery format also align with recent evidence that individuals with MCI and even dementia still frequently use technologies such as smartphones and tablets [57, 72]. These key findings extend limited evidence about preferred intervention features [54], by demonstrating that those suitable for a general older adult population seem likely to be engaging and acceptable for those with lower cognitive performance scores too. These preliminary findings will be explored further in the feasibility trial of the Active Brains intervention [70].

Overall, our findings largely concur with the literature, PPI input and co-investigator expertise that informed our preliminary intervention guiding principles and behavioural analysis, and so largely confirmed the priorities for intervention functions and features. The qualitative data also provide valuable, detailed feedback that has informed the optimisation of Active Brains to maximise the likelihood of intervention engagement and effectiveness. Whilst we will continue to explore these perceptions and experiences in relation to the Active Brains intervention, these tentative insights may be of interest more widely to those developing behaviour change interventions for older adults.

This paper addresses numerous calls to more clearly articulate the intervention development process, and the resulting intervention’s expected mechanisms of action [42]. It is important to test these mechanisms to provide new evidence about behavioural determinants and the most effective intervention functions to target them [42], particularly in multi-domain interventions. This information enables advancement of theoretical understandings of behaviour change in diverse contexts [73]. The extensive qualitative research is a further strength of this rigorous intervention development process. The qualitative interviews provided in-depth understanding of target users’ preferences and life-context to maximise acceptability. These participants represent a wide-ranging community-based group, purposively sampled from JDR as well as primary care. As participants’ understandings of cognitive health were sought during the period that they had access to Active Brains, their views could have been influenced by their experience of the intervention. However, these questions explored perceptions of cognitive health in a broad sense rather than how they related to specific intervention content. Indeed, participants’ responses more often involved accounts of their own beliefs and experiences than reference to Active Brains.

With the development of Active Brains now complete, the next step is a feasibility trial (*n* = 360, in progress) to test the intervention and trial procedures. A fully powered trial (*n* = 20,000) then aims to determine whether the Active Brains intervention can successfully reduce the incidence of dementia amongst older adults over a 5-year period. This systematic development process has closely informed the planning of our later process evaluation that will occur alongside the fully powered effectiveness trial. Specifically, it has helped to identify the intervention’s anticipated mechanisms of action (outlined in the logic model) and, in turn, has informed the inclusion of specific process measures that will allow us to test these mechanisms. This includes measures of self-efficacy, social support, intrinsic motivation for target behaviours and perceptions of intervention usability.

## Conclusions

This study has begun to address the need for a rigorously-developed, low-cost, multi-domain behaviour change intervention for maintaining older adults’ cognitive health. It presents the theory-, evidence- and person-based framework that arose from the planning of the intervention, as well as the primary qualitative evidence that helped to optimise acceptability of intervention content and functionality. As well as facilitating optimisation of intervention content, the qualitative data contribute a greater understanding of older adults’ perceptions of brain health, and the barriers and facilitators to engaging in preventative behaviours. In doing so, this study has provided preliminary evidence that a digitally delivered intervention with minimal support appears acceptable and potentially engaging to older adults with higher and lower levels of cognitive performance.

## Supplementary Information

**Additional file 1:.** Additional Table 1 (.pdf) - Intervention actions employed in each phase of the Active Brains development process

**Additional file 2:.** Additional Table 2 (.pdf) - Search strategies employed in reviewing conducted during development phase

**Additional file 3:.** Additional Table 3 (.pdf) - Excerpts from Table of Changes

**Additional file 4:.** Additional Table 4 (.pdf) - Behavioural analysis of Active Brains using the Behaviour Change Wheel (BCW) and Theoretical Domains Framework (TDF)

**Additional file 5:.** Additional Figure 1 (.jpg) - Full Active Brains intervention logic model

## Data Availability

The datasets used and/or analysed during the current study are available from the corresponding author on reasonable request. Further examples and extracts from data/analysis are available in additional files.

## Abbreviations

WHO: World Health Organisation
MCI: Mild cognitive impairment
AACD: Age-associated cognitive decline
PPI (representative): Public and Patient Involvement (representative)
BCW: Behaviour change wheel
TDF: Theoretical domains framework
BCTs: Behaviour change techniques
MRC: Medical Research Council
JDR: Join Dementia Research
CARE (approach): Congratulate, Ask, Reassure, Encourage (approach)
