# Supplementary material for: Planning and optimising a digital intervention to protect older adults’ cognitive health
Source: Pilot Feasibility Stud. 2021 Aug 18;7:158. doi: 10.1186/s40814-021-00884-2 (PMC8371874; doi:10.1186/s40814-021-00884-2)
Supplement: Supplementary file 1 — Additional file 1:. Additional Table 1 (.pdf) - Intervention actions employed in each phase of the Active Brains development process [file 40814_2021_884_MOESM1_ESM.pdf]

Additional Table 1. Intervention actions employed in each phase of the Active Brains development process

**Phase of Active Brains Development**	**Intervention development actions as identified by O'Cathain, Croot (1)**
Literature reviewing and consultation with multidisciplinary team	1.Identify there is a problem in need of a new intervention<br>2.Establish and group/set of groups to guide the development process<br>3.Understand the problems/issues to be addressed<br>4. Make a decision about the specific problem/problems that intervention will address<br>5.Identify possible ways of making changes to address the problems
Guiding principles	4. Make a decision about the specific problem/problems that an intervention will address<br>5.Identify possible ways of making changes to address the problems<br>7. Consider real-world issues about cost and delivery of intervention<br>9. Generate ideas about solutions and components and features of intervention<br>10. Revisit decision about where to intervene<br>11. Make decisions about format/content and delivery of the intervention
Behavioral analysis and logic model	3.Understand the problems/issues to be addressed<br>5.Identify possible ways of making changes to address the problems<br>6. Specify who will change, how and when<br>7. Consider real-world issues about cost and delivery of intervention<br>9. Generate ideas about solutions and components and features of intervention<br>10. Revisit decision about where to intervene<br>11. Make decisions about format/content and delivery of the intervention<br>12. Design an implementation plan<br>17. Document the intervention
Think aloud interviews	13. Make prototypes or mock-ups of the intervention<br>14. Test on small samples for feasibility and acceptability<br>16. Optimize the intervention for efficiency prior to RCT
Longitudinal interviews	14. Test on small samples for feasibility and acceptability<br>15. Test on a more diverse population<br>16. Optimize the intervention for efficiency prior to RCT

1. O'Cathain A, Croot L, Sworn K, Duncan E, Rousseau N, Turner K, et al. Taxonomy of approaches to developing interventions to improve health: a systematic methods overview. Pilot and feasibility studies. 2019;5(1):41.
