# Supplementary material for: Planning and optimising a digital intervention to protect older adults’ cognitive health
Source: Pilot Feasibility Stud. 2021 Aug 18;7:158. doi: 10.1186/s40814-021-00884-2 (PMC8371874; doi:10.1186/s40814-021-00884-2)
Supplement: Supplementary file 2 — Additional file 2:. Additional Table 2 (.pdf) - Search strategies employed in reviewing conducted during development phase [file 40814_2021_884_MOESM2_ESM.pdf]

Additional Table 2. Search strategies employed in reviewing conducted during development phase

		Search Topic			
		**Digital cognitive training interventions for older adults (OAs)**	**Digital physical activity (PA) interventions for OAs**	**(Digital) PA intervention for cog. impaired OAs**	**(Digital) Sedentary behaviour interventions for OAs**
**Search date**		Spring 2017	17/05/17	06/06/17	12/06/17
**Search Terms**	1.Technology terms	ts=(computer* OR "at home" OR online OR internet OR e-health OR ehealth OR "home deliver*" OR "self initiated" OR "self led" OR home NEAR/5 computer)	ti=(technolog* OR "video*" OR "CD-rom*" OR computer* OR podcast* OR www OR telephone* OR telemed* OR "tele med*" OR "tele-med*" OR telehealth OR "tele health" OR "tele-health" OR "communication technolog*" OR "information technolog*" OR "information-technolog*" OR ict OR "electronic health" OR "electronic-health" OR ehealth OR "e-health" OR internet* OR online* OR "on-line" OR "world wide web" OR "world-wide web" OR email* OR "e-mail*" OR website* OR "web-site*" OR "web-based" OR ipad* OR iOS OR "mobile health" OR mhealth OR "m-health" OR "digital" OR phone* OR "short message service" OR sms OR "text messag*" OR "multimedia messaging service" OR "picture messag*" OR mms OR "digital photograph*" OR app OR apps OR "mobile application*" OR multimedia OR PDA OR "personal digital assistant*" OR "handheld computer*" OR "hand-held computer*" OR "tablet PC" OR "tablet computer*" OR smartphone* OR "smart phone*" OR "smart-phone*" OR iphone* OR "mobile phone*" OR "mobile-phone*" OR "cell-phone*" or "cell phone" OR android OR tracker* OR wearable* OR "activity monitor*" OR fitbit* OR "apple watch" OR "mio fuse" OR "mio alpha" OR "mio link" OR "samsung gear" OR withings OR "misfit shine" OR "misfit flash" OR "jawbone up" OR "nike fuel band" OR "basis band" OR "garmin vivo" OR "social media*" OR blog* OR facebook* OR twitter OR tweet* OR youtube OR whatsapp OR Instagram OR "pokemon go" OR JITAI OR "just-in-time adaptive intervention*")		
	2. Older adult terms	ti=(elder* OR “old* adults” OR “old* people” OR geriatric OR old* OR age* OR senior* OR “senior adults*” OR “senior persons” OR “senior people” OR pension* OR retire* OR “retired adult*” OR “retired people”)	ti=("older adult*" OR elderl* OR senior* OR retir* OR "older people" OR "older person" OR age* OR pension* OR "mature adult*")		
	3. Cognitive training terms	ti=(“brain train*” OR brain-train OR “cognitive train*” OR “cognitive exercise*” OR “brain fit*” OR “brain program*” OR “cognitive program*” OR “brain intervention*” OR “cognitive intervention*” OR “cogniti* intervention*” OR “verbal memory” OR memory OR “working memory” OR “spatial memory” OR “processing speed” OR “speed of processing” OR “executive funct*” OR attention OR attention* OR			

	Search Topic			
	**Digital cognitive training interventions for older adults (OAs)**	**Digital physical activity (PA) interventions for OAs**	**(Digital) PA intervention for cog. impaired OAs**	**(Digital) Sedentary behaviour interventions for OAs**
**Search date**	Spring 2017	17/05/17	06/06/17	12/06/17
	visuospatial OR visuo-spatial OR language OR “verbal fluency”)			
4. Physical activity terms		ti=("physical activit*" OR "motor activit*" OR "outdoor activit*" OR exercis* OR "active transport*" OR "active living" OR "leisure activit*" OR fitness OR acceleromet* OR walk* OR lifestyle* OR "behavio* change*" OR "behavio* intervent*" OR sport*)		
5. Cognitive impairment terms			ti=(cogniti* OR brain* OR alzheim* OR dement* OR memory OR "executive funct*" OR impair*)	
6. Sedentary behaviour terms				ti=(sedentar* OR sit OR sitting)
**COMBINED TERMS**	**1 AND 2 AND 3**	**1 AND 2 AND 4**	**(1) AND 2 AND 4 AND 5**	**(1) AND 2 AND 6**
**Matches returned**[a]	**9,230**	**96**	**(11)<br>602**	**(5)<br>476**

[a] This reports total number of matches, some searches were later filtered by article type (e.g. reviews only)
