# Supplementary material for: Planning and optimising a digital intervention to protect older adults’ cognitive health
Source: Pilot Feasibility Stud. 2021 Aug 18;7:158. doi: 10.1186/s40814-021-00884-2 (PMC8371874; doi:10.1186/s40814-021-00884-2)
Supplement: Supplementary file 3 — Additional file 3:. Additional Table 3 (.pdf) - Excerpts from Table of Changes [file 40814_2021_884_MOESM3_ESM.pdf]

Additional Table 3. Excerpts from Table of Changes

Example from Introductory/linking pages – appearance/ layout of menu pages					
Negative Comments<br><br>Busy/cluttered nature of menu pages makes them difficult to navigate<br><br>"I immediately look at this page and find it untidy and as a, not a struggle, but as a barrier there to reading it clearly and understanding it. I'm struggling to find what to click to go to next. It's not immediately clear." (P0245)<br><br>***<br>"My first thing is, it looks a bit busy. What I think could be good is that you bar- you can click on, 'My Goals' I think that you should pinpoint that they're at the bottom of the page. Cause it's quite a busy page. I think there's a lot of information to take in there." (P0142)	Positive Comments	Possible Change<br><br>Reduce text on page to essential messages only	Reason for change<br><br>REP, EAS	Agreed change<br><br>Reduce amount of text on page; bullet points where possible; add images to break up text	MoScoW<br><br>S
Example from Getting Active Module – activity goal setting					
Negative Comments<br><br>Options to select from in goal setting pages not perceived to be specific enough<br><br>"Okay… Doesn't say here how much…You're planning to… to try and do… I think it should be in terms of… either length of time or… Number of times a week or, or… whatever. It just, you know, setting a goal to walk. Walking, yeah… Yeah, I think there needs to be…You know, for it to feel like a goal for me…It should say, you know, walk so many miles or	Positive Comments	Possible Change<br><br>Give examples of personal goals they can write in.<br><br>Add duration component<br><br>More specific activity options in	Reason for change<br><br>IMP, REP, EXP – more specific (e.g. SMART) goals more likely to be effective	Agreed change<br><br>Make activity options to choose from more specific ; add (optional) planning elements to goal setting – duration, frequency	MoScoW<br><br>M

whatever…Yeah, I can see it's still only saying 'number of days'." (J0103)<br><br>***<br>"I'm sitting there thinking are these specific enough? […] Activities at home is a bit vague, if you don't mind me saying so… You know, or just walk to the shops. Having a little task like that would be more useful than just, say, walking." (P0111)		the drop down menu			
Example from Strength and Balance Module – concerns about safety pages					
**Safety information:**<br><br>**Users don't feel that seeking advice from doctor is reasonable use of GP time, so don't agree with advice given**<br><br>"It's… I always find this information about checking with your doctor before you start interesting, because I very… well, I say that. I very seldom make plans to go and visit the doctor. And I certainly wouldn't regarding this, I think." (P0105)<br><br>***<br><br>There's lots of things I've seen recently, that have 'check with your doctor first.' On the one hand, the television is showing us a surgery waiting room full of people. Who probably are poorly, really poorly. And you've just popped up and said, "Will it be all right if I do some exercise?" (Laughs.)" (P0126)		Change text so that it suggests discussing with family/friends first	**IMP, EAS, EXP** - Agrees with feedback from GP co-I that GP may not be first port of call.	Changed text so that it suggests discussing with family/friends first and then seeking GP advice if still concerned.	

Example from Breaks from Sitting Module – suggestions about ways to break up sedentary time					
**Negative Comments**<br><br>**Activities involving leaving commonly used objects further away not considered realistic**<br><br>"'Leave your bedtime book on the kitchen table'. I don't get that one. How does that work? You're just gonna pick it up when you're downstairs and carry it to the bedroom and then bring it back down…" (P101)<br><br>***<br>"I can see a point about them, but it's probably something that you would do one time and then not do again sort of thing, I wouldn't mind get—you know, I'm only perhaps thinking of myself, but you know… I mean these are, leaving your phone in the hall, you've always got your mobile on you, haven't you?" (P0122)	**Positive Comments**	**Possible Change**<br><br>Remove these suggestions<br><br>Replace with different type of suggestion	**Reason for change**<br><br>**REP, EAS, IMP**	**Agreed change**<br><br>Replace suggestions with recommendations about building low intensity activities into otherwise sedentary time	**MoScoW**<br><br>**M**
Example from Brain Training Module – previous user stories					
**Negative Comments**<br><br>"It's not something that would interest me. I'm sure there are people it would interest but me, not interested." "Nah. I think it's one of those things, you've said yourself, you're gonna do this Brain Training, the only reason you're gonna do it is because you're convinced that it'll possibly help you in some way or another, so you're already convinced" (P0111)	**Positive Comments**<br><br>"Definitely, yes. Yes, again it's interesting to see just the different way people think about it and how to get their target, so yes the	**Possible Change**<br><br>Could consider removing/reducing stories?	**Reason for change**<br><br>**NC**	**Agreed change**<br><br>No action required – mixture of positive and negative feedback indicates more of a personal preference issue.	**MoScoW**<br><br>**n/a**

	stories are good." P0125			Stories are also an optional element	
Example from Eat for Health Module – novelty of information presented					
**Negative Comments**<br><br>"Well, it's giving more and more information, which most people should know, and I certainly do know. I looked at it and I thought: Well, there's nothing here that I'm not aware of or can't do." (P0250)<br><br>"So, I looked at it [Eat for Health session] and I thought, 'Well, there's nothing here that I don't… I'm not aware of, or can't do.'" (P0127)	**Positive Comments**<br><br>"Yeah. That… that just reinforced what I, what I'm doing, which actually gave me some pleasure because, "Oh, yeah, well at least I'm doing the right thing there." So, if you like, that helped." (J0127)<br><br>"It just brings to your mind what you should be doing as opposed to what you are doing in your everyday life, doesn't it?... that's very positive because everyone knows that if you look at a plate of biscuits or some fruit, you should go to the fruit and not	**Possible Change**<br><br>Include content that may be more novel and interesting to users whilst retaining the key underlying 'healthy eating' message	**Reason for change**<br><br>REP, IMP	**Agreed change**<br><br>Move some of the brain-training specific healthy eating content (more likely to be novel) to the start of the session so that people view this content first	**MoScoW**<br><br>S

	the plate of biscuits but your brain tells you, 'I really want the sugar.' So from that, it just focuses you on that sort of thing really" (P0269)				

Coding framework		
**Code**	**Stands for**	**Means**
IMP	Important for behaviour change	This is an important change that is likely to impact behaviour change or a precursor to behaviour change (e.g. acceptability, feasibility, persuasiveness, motivation, engagement), and/or is in line with the Logic Model, and/or is in line with the Guiding Principles For example, participants appear unconvinced by an aspect of the intervention, so you decide to add motivational examples.
EAS	Easy and uncontroversial	An easy and feasible change that doesn't involve any major design changes. For example, a participant was unsure of a technical term, so you add a definition.
REP	Repeatedly	This was said repeatedly, by more than one participant.
EXP	Experience	This is supported by experience. Please specify what kind of experience, for example:<br>1. PPIs agree this would be an appropriate change.<br>2. Experts (e.g. clinicians on your development team) agree that this would be an appropriate change.<br>3. Literature: This is supported by evidence in the literature.
NCON	Does not contradict	This does not contradict experience (e.g. evidence), or the Logic Model, or the Guiding Principles
NC	Not changed	It was decided not to make this change. Please explain why (e.g. it would not be feasible; or only one person said this).
