# Supplementary material for: Planning and optimising a digital intervention to protect older adults’ cognitive health
Source: Pilot Feasibility Stud. 2021 Aug 18;7:158. doi: 10.1186/s40814-021-00884-2 (PMC8371874; doi:10.1186/s40814-021-00884-2)
Supplement: Supplementary file 4 — Additional file 4:. Additional Table 4 (.pdf) - Behavioural analysis of Active Brains using the Behaviour Change Wheel (BCW) and Theoretical Domains Framework (TDF) [file 40814_2021_884_MOESM4_ESM.pdf]

Additional Table 4. Behavioural analysis of Active Brains using the Behaviour Change Wheel (BCW) and Theoretical Domains Framework (TDF)

Target Behaviour	Barrier/ *facilitator* to target behaviour	Evidence for barrier/ facilitator/ intervention ingredient	Intervention ingredient	Target construct (BCW)	Key TDF domains relevant	Intervention function (BCW)	BCT (using 93 BCTs on BCT taxonomy v1)
	**Key behaviour: Initial engagement with Active Brains starter session**						
**Registering on Active Brains study website**	Low health literacy<br>Low digital literacy<br>Busy<br>Too little time<br>Forgetting to do it<br>Privacy and credibility concerns<br><br>*Access to home computer*<br>*Skills in using computer*<br>*Desire to maintain healthy cognitive function*<br>*Time to engage*<br>*Perceived utility of online technology*<br>*Interest in study*	Ware, Bartlett (1) – qual. study of older adults' preferences, interests and concerns re. use of e-health technologies<br><br>Adams, Stubbs (2) – psychological barriers to internet use<br><br>Bozoki, Radovanovic (3) – lack of access a barrier<br><br>O'Connor, Hanlon (4) – factors affecting patient/public engagement with digital health interventions<br><br>PPI provided various suggestions about wording clarification on recruitment materials	Targeting user group who may have more time to engage (retired older adults)<br><br>Persuasive information leaflet highlighting benefits of study<br><br>Information leaflet and website written in clear simple language to ensure readability (iterated with PPI and users)	Psychological capability<br><br>Physical opportunity<br><br>Reflective motivation	Knowledge<br><br>Environmental context and resources<br><br>Beliefs about capabilities, beliefs about consequences, intentions	Education<br>Persuasion<br>Enablement	1.2 Problem Solving<br>5.1 Information about health consequences<br>9.1 Credible source
**Logging onto Active Brains**	Low health literacy<br>Low digital literacy<br>Busy<br>Too little time	Ware, Bartlett (1) – qual. study of older adults' preferences, interests and concerns	Simple login procedures with instructions (iterated with PPI and users)	Physical capability	Skills	Education<br>Enablement<br>Environmental restructuring	3.1 Social support (unspecified)<br>4.1 Instruction on how to perform a behaviour

**starter section**	Forgetting to do it<br><br>*Access to home computer*<br>*Skills in using computer*<br>*Desire to maintain healthy cognitive function*<br>*Time to engage*<br>*Prompts/reminders*<br>*Perceived utility of online technology*<br>*Interest in study*	re. use of e-health technologies<br><br>Adams, Stubbs (2) – psychological barriers to internet use<br><br>Bozoki, Radovanovic (3) – lack of access a barrier	Easily navigable website (iterated with PPI and users)<br><br>Automated reminders if user has not logged in after registration<br><br>Behavioural facilitation (support arm)	Psychological capability<br><br>Automatic motivation<br><br>Physical opportunity<br><br>Social opportunity	Knowledge, memory, attention and decision making processes<br><br>Reinforcement<br><br>Environmental context and resources<br><br>Social influences	Persuasion	7.1 Prompts/cues<br>15.1 Verbal persuasion about capability
**Selecting physical activity, reducing sedentary time, strength and balance, brain training, or healthy eating session in Active Brains website**	Lack of interest in/recognition of importance of behaviour<br>Negative attitudes towards/perceptions of behaviour<br>Behaviour not considered important/relevant<br><br>*Positive outcome expectancy*<br>*Interest in/enjoyment of target behaviour*<br>*Novelty of activities*<br>*Perceived benefits*	van Uffelen, Heesch (5) -people don't accurately recognise/report sedentary time<br><br>Chong, Doyle (6), Cox, Flicker (7) – enjoyment of activities important<br><br>Yardley, Morrison (8) – user preferences for information provision<br><br>PPI feedback suggested some clarifications needed to navigation of 'menu' pages	Introductory section presents rationale for study and different components<br><br>Staggered release of modules to allow gradual engagement with content and periodic 'new' content<br><br>Users presented with choice of sessions, with guided advice tailored to mobility<br><br>Motivational emails highlighting benefits of target behaviours<br><br>Behavioural facilitation (support arm)	Psychological capability<br><br>Physical opportunity<br><br>Social opportunity	Knowledge<br><br>Environmental context and resources<br><br>Social influences	Education<br>Enablement<br>Environmental restructuring<br>Modelling<br>Persuasion	3.1 Social support (unspecified)<br>5.1 Information about health consequences<br>5.3 Information about social and environmental consequences<br>5.6 Information about emotional consequences<br>7.1 Prompts/cues<br>9.1 Credible source<br>15.1 Verbal persuasion about capability

	Key behaviour: Increased physical activity						
**Setting goals to increase physical activity**	Process too complicated<br>Suggested goals/activities not considered relevant<br>Low self-efficacy for activities<br><br>*Perceived credibility/trustworthiness/ persuasiveness*<br>*Ease of process*<br>*Desire to challenge self*<br>*Desire to improve fitness*<br>*Activities seem achievable/realistic*<br>*Self-efficacy for activities*<br>*Perceived benefits*	Gray, Hunt (9) – simplicity of activities appreciated<br><br>Erickson, Voss (10)<br>Harris, Kerry (11)<br>Marcus, Forsyth (12)<br>Orbell and Sheeran (13)<br>Nyman, Adamczewska (14)- evidence of effectiveness of specific physical activities or activity programmes or techniques for initiating new behaviours (e.g. goal setting)<br><br>Aalbers, Baars (15), Müller and Khoo (16), Peels, van Stralen (17) – tailoring for different levels of capability important<br><br>Price, Corwin (18) - evidence-based, credible communication of link between increasing physical activity and cognitive health	Content focused on building motivation and capability to engage in physical activity through:<br>- explanation of key benefits backed up by evidence;<br>- suggestion of simple, graded activities, tailored to mobility/current physical activity level – walking recommended as primary activity;<br>- examples of others and their experiences;<br>- structured guidance on simple goal setting and action planning;<br>- addressing concerns that users may have<br><br>Pedometers available for individuals to self-monitor and set goals related to step count<br><br>Motivational emails highlighting benefits of physical activity and reminding users that they can set/revise goals at any time	Psychological capability<br><br>Reflective motivation<br><br>Physical opportunity<br><br>Social opportunity	Behavioural regulation<br><br>Goals, beliefs about capabilities, optimism, intentions, beliefs about consequences<br><br>Environmental context and resources<br><br>Social influences	Education<br>Enablement<br>Environmental restructuring<br>Modelling<br>Persuasion	1.1 Goal setting (behaviour)<br>1.2 Problem Solving<br>1.4 Action Planning<br>3.1 Social support (unspecified)<br>4.1 Instruction on how to perform a behaviour<br>4.2 Information about antecedents<br>5.1 Information about health consequences<br>5.3 Information about social and environmental consequences<br>5.6 Information about emotional consequences<br>7.1 Prompts/cues<br>8.2 Behaviour substitution<br>8.3 Habit formation<br>8.4 Habit reversal<br>8.7 Graded tasks<br>9.1 Credible source<br>11.3 Conserving mental resources<br>12.2 Restructuring the social environment<br>12.5 Adding objects to the environment<br>15.1 Verbal persuasion about capability

**Engaging in increased physical activity**	Poor health status/ physical functional ability, e.g. frailty, pain, memory problems<br>Low self-efficacy<br>Poor outcome expectations<br>Inactive lifestyle habits<br>Unsuitable environment – safety (fear of falling); availability and accessibility of facilities e.g. parks<br>Inflexible routine<br>Weather<br>Doubts about capabilities<br>Doubts about necessity of PA<br>Negative attitudes towards PA<br>Lack of social support<br>Low motivation<br><br>*Perceived benefits and value of PA*<br>*Fun/Enjoyment*<br>*Sense of control over one's physical activity behaviour*<br>*Social support and maintenance of social bonds*<br>*Prompts/reminders*<br>*Physician advice*<br>*Flexible routine*<br>*Activities perceived as realistic*	**Determinants**<br>Chong, Doyle (6), Forberger, Bammann (19), Greaney, Lees (20), Baert, Gorus (21), Warner, Wolff (22), Rhodes, Martin (23), Direito, Walsh (24), Martin and Sinden (25), Van Dyck, Cardon (26), Smith, Banting (27), Clarke, Sniehotta (28), Manz, Mensink (29), Notthoff, Reisch (30), Devereux-Fitzgerald, Powell (31), Schutzer and Graves (32), Normansell, Smith (33)<br><br>**Intervention ingredients**<br><br>Tang, Smith (34) – information about social, emotional and environmental consequences (BCTs) associated with larger effect sizes for change in self-efficacy for PA<br>Notthoff, Klomp (35) – older adults show greater motivation and better recognition of messages in PA interventions when messages are positively framed	(Also as above)<br>Printable plans available from website, to position somewhere memorable at home<br><br>Motivational emails sent to remind users to engage in activities at user-selected time-points<br><br>'Reasons to stay active' card – with examples from others<br><br>Users encouraged to discuss concerns/ fears/ perceived barriers with friends/ family members and to explain the activities they are participating in so as to allow the individual to provide encouragement and reassurance<br><br>Behavioural facilitation (support arm) - contact with facilitator will provide praise, encouragement, reassurance and will seek to help the participant solve any issues faced	Automatic motivation<br><br>Reflective motivation<br><br>Social opportunity	Reinforcement, emotion<br><br>Goals, beliefs about capabilities, optimism, intentions, beliefs about consequences<br><br>Social influences	Education<br>Environmental restructuring<br>Persuasion	3.1 Social support (unspecified)<br>3.3 Social support emotional<br>4.1 Instruction on how to perform a behaviour<br>5.1 Information about health consequences<br>5.3 Information about social and environmental consequences<br>5.6 Information about emotional consequences<br>7.1 Prompts/cues<br>8.1 Behavioural practice/rehearsal<br>8.3 Habit formation<br>8.7 Graded tasks<br>13.1 Identification of self as role model<br>15.1 Verbal persuasion about capability<br>16.3 Vicarious consequences

		Nyman, Adamczewska (14), Peels, van Stralen (17), Peels, van Stralen (36), Lara, O'Brien (37), Peels, de Vries (38), Peels, Bolman (39) - potentially important components of interventions for increasing PA: self-monitoring; social aspects/support; suggestions for local physical activity options; promotion of autonomy and provision of positive reinforcement for small achievements; tailoring for participants with different limitations; optional exercises; simple goal setting					
**Key behaviour: Reduced sedentary behaviour**							
**Making plans to take breaks from sitting**	Process too complicated<br>Suggested plans not considered relevant/realistic<br>Low self-efficacy for making changes<br><br>*Perceived credibility/trustworthiness/ persuasiveness*<br>*Ease of process*	Gardner, Smith (40) – education and persuasion important components of promising interventions to reduce sedentary time<br><br>Marcus, Forsyth (12), Orbell and Sheeran (13) – evidence for efficacy of goal setting/	Website content focused on building motivation and capability to take breaks from sitting through:<br>- explanation of key benefits backed up by evidence;<br>- suggestion of simple activities, tailored to mobility/current physical activity level;	Psychological capability<br><br>Reflective motivation	Behavioural regulation<br><br>Goals, beliefs about capabilities, optimism, intentions, beliefs about consequences	Education<br>Enablement<br>Environmental restructuring<br>Modelling<br>Persuasion	1.1 Goal setting (behaviour)<br>1.2 Problem Solving<br>1.4 Action Planning<br>1.9 Commitment<br>3.1 Social support (unspecified)<br>4.1 Instruction on how to perform a behaviour<br>4.2 Information about antecedents

	Desire to challenge self<br>*Desire to improve fitness*<br>*Changes seem achievable/realistic*<br>*Self-efficacy for making changes*<br>*Perceived benefits*	implementation intentions in increasing PA<br><br>Aalbers, Baars (15), Müller and Khoo (16), Peels, van Stralen (17) – tailoring for different levels of capability important<br><br>Price, Corwin (18) - evidence-based, credible communication of link between increasing physical activity and cognitive health	- examples of others and their experiences;<br>- structured guidance on goal setting and planning actions at specific times/in specific contexts;<br>- addressing concerns that users may have<br><br>Motivational emails highlighting benefits of taking breaks from sitting and reminding users that they can set/revise goals at any time	Physical opportunity<br><br>Social opportunity	Environmental context and resources<br><br>Social influences		5.1 Information about health consequences<br>5.3 Information about social and environmental consequences<br>5.6 Information about emotional consequences<br>7.1 Prompts/cues<br>8.2 Behaviour substitution<br>8.3 Habit formation<br>8.7 Graded tasks<br>9.1 Credible source<br>11.3 Conserving mental resources<br>15.1 Verbal persuasion about capability
**Modifying physical environment to encourage breaks from sitting**	Lack of knowledge about how to implement<br>Considered too much effort to implement changes<br>Suggested changes not considered relevant/realistic<br><br>*Perceived credibility/trustworthiness/ persuasiveness*<br>*Ease of making changes*<br>*Changes seem achievable/realistic*<br>*Perceived benefits*	Gardner, Smith (40) – environmental restructuring (physical and social) an important component of promising interventions to reduce sedentary time<br><br>PPI feedback on early material question realistic nature of some of the action planning suggestions	As part of goal setting, users are given information about the value of modifying the physical environment to encourage behaviour change and ideas as to how to do it<br><br>Action planning offers suggestions of simple and quick ways to do this that users can choose from when setting goals	Reflective motivation<br><br>Physical opportunity	Goals, intentions, beliefs about consequences<br><br>Environmental context and resources	Education<br>Environmental restructuring<br>Modelling<br>Persuasion	1.4 Action Planning<br>4.1 Instruction on how to perform a behaviour<br>5.1 Information about health consequences<br>5.3 Information about social and environmental consequences<br>5.6 Information about emotional consequences<br>7.1 Prompts/cues<br>8.2 Behaviour substitution<br>8.3 Habit formation<br>12.1 Restructuring the physical environment<br>12.2 Restructuring the social environment
**Taking breaks from sitting**	Pain/stiffness,<br>Low self-efficacy	Chastin, Fitzpatrick (41), Chastin, Buck (42), Owen, Sugiyama (43),	(Also as per 'making plans' above)	Automatic motivation	Reinforcement	Education<br>Environmental restructuring	3.1 Social support (unspecified)

	Functional limitations<br>Ageist stereotypes/perceptions<br>Attractiveness/ enjoyment of sedentary activities<br>Low control over behaviour (e.g. at work)<br>Retirement status<br>Perceived safety of neighbourhood<br>Availability of places to rest<br><br>*Pain/stiffness*<br>*Self-efficacy*<br>*Desire to relieve boredom*<br>*Perception of tasks to do*<br>*Family and social commitments and*<br>*Motivation,*<br>*Good weather,*<br>*Desire to show independence*<br>*Improved mood from movement*	Brug and Chinapaw (44) - determinants of sedentary behaviour<br><br>Gardner, Smith (40)– review of sedentary behaviour intervention components<br><br>Van Dyck, Cardon (26) - psychological, social and physical environmental characteristics predicting changes in physical activity and sedentary behaviours	Printable plans available from website, to position somewhere memorable at home<br><br>Motivational emails sent to remind users to engage in activities at user-selected time-points<br><br>Behavioural facilitation (support arm) -contact with facilitator will provide praise, encouragement, reassurance and will seek to help the participant solve any issues faced	Reflective motivation<br><br><br><br><br><br><br><br>Social opportunity	Goals, beliefs about capabilities, optimism, intentions, beliefs about consequences<br><br><br><br><br>Social influences	Persuasion	4.1 Instruction on how to perform a behaviour<br>5.1 Information about health consequences<br>5.3 Information about social and environmental consequences<br>5.6 Information about emotional consequences<br>7.1 Prompts/cues<br>8.1 Behavioural practice/rehearsal<br>8.3 Habit formation<br>8.7 Graded tasks<br>13.1 Identification of self as role model<br>13.2 Framing/reframing<br>15.1 Verbal persuasion about capability
	**Key behaviour: Uptake of strength and balance exercises**						
**Setting goals/ action plans to do exercises**	Process too complicated<br>Suggested exercises not considered relevant/realistic<br>Low self-efficacy for exercises<br>Perceived lack of time to do exercises<br>Concerns about whether exercises are safe	Fiatarone Singh, Gates (45) – evidence for effectiveness of strength training in protecting cognitive health<br><br>Clemson, Fiatarone Singh (46), Clemson, Munro (47) – evidence for effectiveness of S & B programme with	Website content focused on building motivation and capability to engage in strength and balance exercises through:<br>- encouraging that simple exercises are built into daily activities<br>- explanation of key benefits backed up by evidence;	Psychological capability<br><br>Reflective motivation<br><br><br><br><br><br>Physical opportunity	Behavioural regulation<br><br>Goals, beliefs about capabilities, optimism, intentions, beliefs about consequences	Education<br>Enablement<br>Environmental restructuring<br>Modelling<br>Persuasion	1.1 Goal setting (behaviour)<br>1.2 Problem Solving<br>1.4 Action Planning<br>1.9 Commitment<br>3.1 Social support (unspecified)<br>3.3 Social support (emotional)<br>4.1 Instruction on how to perform a behaviour<br>4.2 Information about antecedents

	Perceived credibility/trustworthiness/ persuasiveness<br>*Ease of process*<br>*Desire to improve strength and balance*<br>*Exercises seem achievable/realistic*<br>*Self-efficacy for exercises*<br>*Perceived benefits*	exercises built into daily activities<br><br>Marcus, Forsyth (12), Orbell and Sheeran (13) – evidence for efficacy of goal setting/ implementation intentions in increasing PA<br><br>Aalbers, Baars (15), Müller and Khoo (16), Peels, van Stralen (17) – tailoring for different levels of capability important<br><br>Price, Corwin (18) - evidence-based, credible communication of link between increasing physical activity and cognitive health	- suggestion of simple activities, tailored to mobility/current physical activity level;<br>- examples of others and their experiences;<br>- structured guidance on goal setting/ action planning;<br>- addressing concerns that users may have		Environmental context and resources		5.1 Information about health consequences<br>5.6 Information about emotional consequences<br>6.1 Demonstration of the behaviour<br>7.1 Prompts/cues<br>8.2 Behaviour substitution<br>8.3 Habit formation<br>8.7 Graded tasks<br>9.1 Credible source<br>11.3 Conserving mental resources<br>12.1 Restructuring the physical environment<br>12.5 Adding objects to the environment<br>13.2 Framing/reframing
**Doing strength and balance exercises**	Poor health status/ physical functional ability, e.g. frailty, pain, memory problems<br>Low self-efficacy for exercises<br>Poor outcome expectations<br>Safety concerns (fear of falling)<br>Doubts about necessity of exercises<br>Lack of social support<br>Low motivation	Share many of the same determinants as general increase in PA behaviour, e.g.:<br><br>Chong, Doyle (6), Forberger, Bammann (19), Greaney, Lees (20), Baert, Gorus (21), Warner, Wolff (22), Rhodes, Martin (23), Direito, Walsh (24), Martin and Sinden (25), Van Dyck, Cardon (26),	(Also, as above)<br><br>Audio-visual demonstration of the exercises to refer back to<br><br>Printable plans available from website, to position somewhere memorable at home<br><br>Motivational emails highlighting benefits of strength and balance	Physical capability<br><br>Automatic motivation<br><br>Reflective motivation	Physical skills<br><br>Reinforcement<br><br>Goals, beliefs about capabilities, optimism, intentions, beliefs about consequences	Education<br>Environmental restructuring<br>Persuasion<br>Training	3.3 Social support (emotional)<br>4.1 Instruction on how to perform a behaviour<br>5.1 Information about health consequences<br>6.1 Demonstration of the behaviour<br>7.1 Prompts/cues<br>8.1 Behavioural practice/rehearsal<br>8.3 Habit formation<br>8.7 Graded tasks

	Perceived benefits and value of strength and balance<br>*Fun/Enjoyment*<br>*Sense of control over one's physical capability*<br>*Social support*<br>*Prompts/reminders*<br>*Physician advice*<br>*Exercises perceived as realistic*	Smith, Banting (27), Clarke, Sniehotta (28), Manz, Mensink (29), Notthoff, Reisch (30), Devereux-Fitzgerald, Powell (31), Schutzer and Graves (32), Normansell, Smith (33)	exercise and reminding users that they can set/revise goals at any time<br><br>Users encouraged to discuss concerns/ fears/ perceived barriers with friends/ family members and to explain the activities they are participating in so as to allow the individual to provide encouragement and reassurance<br><br>Behavioural facilitation (support arm) -contact with facilitator will provide praise, encouragement, reassurance and will seek to help the participant solve any issues faced	Social opportunity	Social influences		12.5 Adding objects to the environment<br>15.1 Verbal persuasion about capability
	**Key behaviour: Uptake of brain training**						
**Making a plan to brain train regularly**	Process too complicated<br>Brain Training games not considered relevant<br>Low self-efficacy for brain training games<br><br>*Perceived credibility/trustworthiness/ persuasiveness*<br>*Ease of process*<br>*Desire to challenge self*<br>*Desire to keep brain active*<br>*Self-efficacy for BT games*<br>*Perceived benefits*	Bozoki, Radovanovic (3) – willingness to challenge self impacts on engagement with online cognitive training<br><br>Haesner, Steinert (48) – perceived utility/ outcome expectations important for engagement with online cognitive training	Website content focused on building motivation and capability to engage in brain training through:<br>- explanation of key benefits backed up by evidence;<br>- examples of others and their experiences;<br>- structured guidance on planning/goal setting;<br>- addressing concerns that users may have	Psychological capability<br><br>Reflective motivation<br><br>Physical opportunity	Behavioural regulation<br><br>Goals, beliefs about capabilities, optimism, intentions, beliefs about consequences<br><br>Environmental context and resources	Education<br>Enablement<br>Environmental restructuring<br>Modelling<br>Persuasion	1.1 Goal setting (behaviour)<br>1.2 Problem Solving<br>1.4 Action Planning<br>3.1 Social support (unspecified)<br>3.3 Social support (emotional)<br>4.1 Instruction on how to perform a behaviour<br>4.2 Information about antecedents<br>5.1 Information about health consequences<br>7.1 Prompts/cues

			Motivational emails highlighting benefits of brain training	Social opportunity	Social influences		8.3 Habit formation<br>8.7 Graded tasks<br>9.1 Credible source<br>13.2 Framing/reframing
**Initial access to, and playing, brain training games**	Low perceived capability/self-efficacy<br>Difficult to access/use<br><br>*Positive outcome expectancy*<br>*Previous experience*<br>*Enjoyment/interest in activities*<br>*Credibility/trustworthiness*	Gates, Sachdev (49), Corbett, Owen (50), Kueider, Parisi (51), Willis, Tennstedt (52) - evidence that online cognitive training effective<br><br>Kueider, Parisi (51) – most older adults didn't need to be tech savvy to benefit from online cognitive training<br><br>McDougall Jr (53), Pike, Chong (54), Mohan (55) – self-efficacy and perceptions of ability to use and complete tasks important	(Also, as above)<br><br>Printable plans available from website, to position somewhere memorable at home<br><br>Motivational emails sent to remind users to engage in brain training at regular intervals	Physical capability<br><br>Reflective motivation<br><br>Physical opportunity	Skills<br><br>Goals, beliefs about capabilities, optimism, intentions, beliefs about consequences<br><br>Environmental context and resources	Education<br>Environmental restructuring<br>Persuasion<br>Training	4.1 Instruction on how to perform a behaviour<br>5.1 Information about health consequences<br>7.1 Prompts/cues<br>8.1 Behavioural practice/rehearsal<br>8.3 Habit formation<br>8.7 Graded tasks
**Regularly logging back in to complete brain training games**	Forgetting<br>Boredom/loss of interest<br>Low perceived capability/self-efficacy<br>Perception of poor previous performance<br>Difficult to access/use<br><br>*Positive outcome expectancy*<br>*Previous experience*<br>*Enjoyment/interest in activities*<br>*Credibility/trustworthiness*	Haesner, O'Sullivan (56) – interest/enjoyment of activities important<br><br>McDougall Jr (53), Pike, Chong (54), Mohan (55) – self-efficacy and perceptions of ability to use and complete tasks important<br><br>Corbett, Owen (50) – loss of engagement high	Motivational emails highlighting benefits of brain training<br><br>New games released over time to keep users stimulated and interested in brain training<br><br>Users encouraged to discuss concerns/ fears/ perceived barriers with friends/ family members and to explain the activities they are	Psychological capability<br><br>Reflective motivation	Memory, attention and decision processes<br><br>Goals, beliefs about capabilities, optimism, intentions, beliefs about consequences	Education<br>Environmental restructuring<br>Persuasion	3.1 Social support (unspecified)<br>3.3 Social support (emotional)<br>5.1 Information about health consequences<br>7.1 Prompts/cues<br>8.1 Behavioural practice/rehearsal<br>8.3 Habit formation<br>8.7 Graded tasks<br>15.1 Verbal persuasion about capability

	Sense of challenge/ competition with self	PPI reported need for more clarity about how long games are likely to take	participating in so as to allow the individual to provide encouragement and reassurance<br><br>Behavioural facilitation (support arm) - contact with facilitator will provide praise, encouragement, reassurance and will seek to help the participant solve any issues faced	Automatic motivation<br><br>Social opportunity	Reinforcement, emotion<br><br>Social influence		
	**Key behaviour: Uptake of Mediterranean diet (plus nuts)**						
**Setting goals to eat more healthily**	Process too complicated<br>Negative attitudes towards/perceptions of healthy eating<br>Healthy eating not considered important/relevant<br>Low self-efficacy for making dietary changes<br><br>*Perceived credibility/trustworthiness/ persuasiveness*<br>*Ease of process*<br>*Positive outcome expectancy*<br>*Perceived benefits – e.g. health, weight loss*<br>*Social support*<br>*Self-efficacy*	Valls-Pedret, Sala-Vila (57) – evidence for med diet as protective of cognitive health<br><br>Gray, Hunt (9) – simplicity of advice about lifestyle behaviour change appreciated	Website content focused on building motivation and capability to eat more healthily through:<br><br>- simple messages about small changes to make for healthier food choices<br>- explanation of key benefits backed up by evidence;<br>- examples of others and their experiences;<br>- structured guidance on goal setting;<br>- addressing concerns that users may have<br><br>Motivational emails highlighting benefits of healthy eating	Psychological capability<br><br>Reflective motivation<br><br>Physical opportunity<br><br>Social opportunity	Behavioural regulation<br><br>Goals, beliefs about capabilities, optimism, intentions, beliefs about consequences<br><br>Environmental context and resources<br><br>Social influences	Education<br>Enablement<br>Environmental restructuring<br>Modelling<br>Persuasion	1.1 Goal setting (behaviour)<br>1.2 Problem Solving<br>1.4 Action Planning<br>1.9 Commitment<br>3.1 Social support (unspecified)<br>3.3 Social support (emotional)<br>4.1 Instruction on how to perform a behaviour<br>4.2 Information about antecedents<br>5.1 Information about health consequences<br>7.1 Prompts/cues<br>8.3 Habit formation<br>8.7 Graded tasks<br>9.1 Credible source<br>12.2 Restructuring the social environment

**Engaging in healthier eating**	Cost<br>Availability of cheap junk food<br>Eating out/ socially<br>Pleasure (esp. alcohol)<br>Dislike of healthy food options<br>Competing priorities/time<br>Unplanned shopping<br>Entrenched food beliefs/ behaviours<br>Identity<br>Lack of capability/ knowledge<br><br>*Clear simple advice, advice around swapping foods*<br>*Experience of ill-health/ health concerns*<br>*Short-term benefits*<br>*Weight loss,*<br>*Identity*<br>*Friends/family support,*<br>*Perceived capability to make dietary changes*	Petroka, Campbell-Bussiere (58), Seguin, Connor (59), Kelly, Martin (60), Nicklett and Kadell (61), Lo, Chang (62), Payette and Shatenstein (63), Bloom, Edwards (64), Bloom, Lawrence (65), Robinson (66), Appleton, Dinnella (67), Sahyoun, Zhang (68), Greene, Lofgren (69) – various determinants of healthy eating behaviour (especially amongst older adults)<br><br>Gray, Hunt (9) – simplicity of advice appreciated	(Also, as above)<br><br>Printable plans available from website, to position somewhere memorable at home<br><br>Motivational emails sent to remind users to engage in activities at user-selected time-points<br><br>Users encouraged to discuss concerns/ fears/ perceived barriers with friends/ family members and to explain the activities they are participating in so as to allow the individual to provide encouragement and reassurance<br><br>Behavioural facilitation (support arm) - contact with facilitator will provide praise, encouragement, reassurance and will seek to help the participant solve any issues faced	Automatic motivation<br><br>Reflective motivation<br><br>Physical opportunity<br><br>Social opportunity	Reinforcement, emotion<br><br>Goals, beliefs about capabilities, optimism, intentions, beliefs about consequence<br><br>Environmental context and resources<br><br>Social influences	Education<br>Environmental restructuring<br>Persuasion	3.3 Social support (emotional)<br>4.1 Instruction on how to perform a behaviour<br>5.1 Information about health consequences<br>7.1 Prompts/cues<br>8.1 Behavioural practice/rehearsal<br>8.3 Habit formation<br>8.7 Graded tasks<br>15.1 Verbal persuasion about capability
	**Key behaviour: Reviewing behaviour and revising goals for all behaviours**						
**Reviewing behaviour and revising goals**	Boredom<br>Perceived poor performance<br>Competing priorities<br>Forgetting<br><br>*Perceived good performance*	Lara, O'Brien (37) - Self-monitoring as important ingredient<br><br>O'Brien, McDonald (70) – feedback appears promising for long-term	Simple, easy to access goal review facility providing tailored feedback<br><br>Motivational emails reminding users to review and revise goals	Psychological capability	Memory, attention and decision processes, behavioural regulation	Education<br>Environmental restructuring<br>Modelling<br>Persuasion	1.1 Goal setting (behaviour)<br>1.2 Problem Solving<br>1.4 Action Planning<br>1.5 Review behaviour goals<br>1.9 Commitment<br>2.2 Feedback on behaviour

	Prompts/reminders<br>*Keen to monitor progress*	engagement with PA behaviours<br><br>Gardner, Smith (40)– review of sedentary behaviour intervention components – self-monitoring important	Goal review website page offering tailored positive feedback on behaviour, links to other activities to try and stories of other users' experiences<br><br>Behavioural facilitation (support arm)	Automatic motivation<br><br>Reflective motivation<br><br><br><br>Physical opportunity<br><br>Social opportunity	Reinforcement, emotion<br><br>Goals, intentions, beliefs about consequence<br><br>Environmental context and resources<br><br>Social influences		2.3 Self-monitoring of behaviour<br>3.1 Social support (unspecified)<br>4.2 Information about antecedents<br>5.1 Information about health consequences<br>5.3 Information about social and environmental consequences<br>5.6 Information about emotional consequences<br>6.2 Social comparison<br>7.1 Prompts/cues<br>8.2 Behaviour substitution<br>8.3 Habit formation<br>8.7 Graded tasks<br>10.4 Social reward<br>11.3 Conserving mental resources<br>12.1Restructuring the physical environment<br>12.2 Restructuring the social environment<br>12.5 Adding objects to the environment<br>15.1 Verbal persuasion about capability<br>15.3 Focus on past success
	**Key behaviour: Integration of Active Brains activities into daily routines**						
**Integrating chosen behaviours into daily life *(reducing***	Forgetting<br>Lack of time/ competing priorities<br>Negative experience of trying new activities<br>Low perceived quality of the intervention<br>Environmental cues	Stiggelbout, Hopman-Rock (71)– predicting maintenance of exercise in older adults<br><br>Brooker, Wesnes (72) – evidence about day to day activities with	Motivational emails to prompt continued engagement with activities and present new ideas outside of website will focus on:	Psychological capability	Memory, attention and decision processes, behavioural regulation	Education<br>Environmental restructuring<br>Modelling<br>Persuasion	1.1 Problem Solving<br>1.5 Review behaviour goals<br>3.1 Social support (unspecified)<br>4.1 Instruction on how to perform a behaviour<br>4.2 Information about antecedents

reliance on Active Brains starter session)	*Social support*<br>*Simplicity of behaviours*<br>*Fit of behaviours with daily life*<br>*Enjoyment of activities*<br>*Experience of benefit from participation*<br>*Enjoyment/ positive [perceptions of intervention*	benefits for cognitive function	- reinforcing key messages about benefits,<br>- suggest new activities or ideas,<br>- prompt continued engagement and behavioural rehearsal/substitution<br><br>Additional content at 7 months will offer:<br><br>- online and offline resources links that might interest users<br>- access to all previous content<br>- ideas about day-to-day activities that can also help keep the brain active<br><br>Behavioural facilitation (support arm)	Automatic motivation<br><br>Reflective motivation<br><br>Physical opportunity<br><br>Social opportunity	Reinforcement, emotion<br><br>Intentions, beliefs about consequences<br><br>Environmental context and resources<br><br>Social influences		5.1 Information about health consequences<br>5.3 Information about social and environmental consequences<br>5.6 Information about emotional consequences<br>7.1 Prompts/cues<br>8.1 Behavioural practice/rehearsal<br>8.2 Behaviour substitution<br>8.3 Habit formation<br>8.6 Generalisation of target behaviour<br>8.7 Graded tasks<br>10.4 Social reward<br>12.1 Restructuring the physical environment<br>12.2 Restructuring the social environment<br>15.1 Verbal persuasion about capability<br>15.3 Focus on past success
**Key behaviour: Maintained engagement with Active Brains intervention**							
**Logging into Active Brains study website**	Forgetting<br>Lack of time<br>Feeling discouraged/ frustrated/ bored of intervention<br><br>*Ease of use*<br>*Enjoyment of website/intervention*<br>*Looking for specific information/advice*	O'Connor, Hanlon (4) – factors affecting patient/public engagement with digital health interventions<br><br>Yardley, Morrison (8) – user preferences for information provision	Motivational emails to remind users to log into website and give reasons for engagement<br><br>Access to new brain training games/ new sections of website contingent on regular access to website	Automatic motivation<br><br>Physical opportunity	Reinforcement, emotion<br><br>Environmental context and resources	Education<br>Environmental restructuring<br>Persuasion<br>Incentivisation	3.1 Social support (unspecified)<br>4.1 Instruction on how to perform a behaviour<br>5.1 Information about health consequences<br>7.1 Prompts/cues<br>9.1 Credible source<br>10.1 Material incentive (behaviour)<br>10.2 Material incentive (reward)

			After 7 months recommendations about a 'maintenance' brain training schedule				
**Completing intervention measures**	Forgetting<br>Lack of time<br>Length of measures<br>Complexity of tasks<br><br>*Altruism*<br>*Perception of benefit*<br>*Ease of participation*	Rolstad, Adler (73), Iglesias and Torgerson (74) – evidence about influences on completion of questionnaires<br><br>PPI reported early draft of follow-up measures being too extensive and time-consuming – decision made to split into small 'essential' and larger 'secondary' measures	Intervention measures limited to only essential measures, clearly explained and introduced to users, suitable for target user group<br><br>Broken into sections to allow completion of 'essential' and secondary measures separately<br><br>Motivational emails to remind users to log into website and give reasons for engagement	Psychological capability<br><br>Automatic motivation<br><br>Physical opportunity	Memory, attention and decision processes<br><br>Reinforcement, emotion<br><br>Environmental context and resources	Education<br>Enablement<br>Environmental restructuring<br>Persuasion	3.1 Social support (unspecified)<br>4.1 Instruction on how to perform a behaviour<br>5.1 Information about health consequences<br>7.1 Prompts/cues<br>9.1 Credible source

N.B. The table provides key examples of evidence about behavioural determinants and intended intervention functions but is not an exhaustive record

References

1. Ware P, Bartlett SJ, Paré G, Symeonidis I, Tannenbaum C, Bartlett G, et al. Using eHealth Technologies: Interests, Preferences, and Concerns of Older Adults. Interact J Med Res. 2017;6(1):e3.
2. Adams N, Stubbs D, Woods V. Psychological barriers to Internet usage among older adults in the UK. Medical informatics and the Internet in medicine. 2005;30(1):3-17.
3. Bozoki A, Radovanovic M, Winn B, Heeter C, Anthony JC. Effects of a computer-based cognitive exercise program on age-related cognitive decline. Archives of Gerontology and Geriatrics. 2013;57(1):1-7.
4. O'Connor S, Hanlon P, O'Donnell CA, Garcia S, Glanville J, Mair FS. Understanding factors affecting patient and public engagement and recruitment to digital health interventions: a systematic review of qualitative studies. BMC Medical Informatics and Decision Making. 2016;16(1):120.
5. van Uffelen JGZ, Heesch KC, Hill RL, Brown WJ. A qualitative study of older adults' responses to sitting-time questions: do we get the information we want? BMC Public Health. 2011;11(1):458.
6. Chong TW, Doyle CJ, Cyarto EV, Cox KL, Ellis KA, Ames D, et al. Physical activity program preferences and perspectives of older adults with and without cognitive impairment. Asia-Pacific Psychiatry. 2014;6(2):179-90.

7. Cox KL, Flicker L, Almeida OP, Xiao J, Greenop KR, Hendriks J, et al. The FABS trial: a randomised control trial of the effects of a 6-month physical activity intervention on adherence and long-term physical activity and self-efficacy in older adults with memory complaints. Preventive medicine. 2013;57(6):824-30.
8. Yardley L, Morrison LG, Andreou P, Joseph J, Little P. Understanding reactions to an internet-delivered health-care intervention: accommodating user preferences for information provision. BMC Medical Informatics and Decision Making. 2010;10(1):52.
9. Gray CM, Hunt K, Mutrie N, Anderson AS, Leishman J, Dalgarno L, et al. Football Fans in Training: the development and optimization of an intervention delivered through professional sports clubs to help men lose weight, become more active and adopt healthier eating habits. BMC Public Health. 2013;13(1):232.
10. Erickson KI, Voss MW, Prakash RS, Basak C, Szabo A, Chaddock L, et al. Exercise training increases size of hippocampus and improves memory. Proceedings of the National Academy of Sciences. 2011;108(7):3017-22.
11. Harris T, Kerry SM, Limb ES, Furness C, Wahlich C, Victor CR, et al. Physical activity levels in adults and older adults 3–4 years after pedometer-based walking interventions: Long-term follow-up of participants from two randomised controlled trials in UK primary care. PLOS Medicine. 2018;15(3):e1002526.
12. Marcus BH, Forsyth LH, Stone EJ, Dubbert PM, McKenzie TL, Dunn AL, et al. Physical activity behavior change: Issues in adoption and maintenance. Health Psychology. 2000;19(1, Suppl):32-41.
13. Orbell S, Sheeran P. Motivational and Volitional Processes in Action Initiation: A Field Study of the Role of Implementation Intentions1. Journal of Applied Social Psychology. 2000;30(4):780-97.
14. Nyman SR, Adamczewska N, Howlett N. Systematic review of behaviour change techniques to promote participation in physical activity among people with dementia. British journal of health psychology. 2018;23(1):148-70.
15. Aalbers T, Baars M, Rikkert MO. Characteristics of effective Internet-mediated interventions to change lifestyle in people aged 50 and older: a systematic review. Ageing research reviews. 2011;10(4):487-97.
16. Müller AM, Khoo S. Non-face-to-face physical activity interventions in older adults: a systematic review. International Journal of Behavioral Nutrition and Physical Activity. 2014;11(1):35.
17. Peels DA, van Stralen MM, Bolman C, Golsteijn RH, de Vries H, Mudde AN, et al. Development of web-based computer-tailored advice to promote physical activity among people older than 50 years. J Med Internet Res. 2012;14(2):e39.
18. Price AE, Corwin SJ, Friedman DB, Laditka SB, Colabianchi N, Montgomery KM. Older Adults' Perceptions of Physical Activity and Cognitive Health: Implications for Health Communication. Health Education & Behavior. 2011;38(1):15-24.
19. Forberger S, Bammann K, Bauer J, Boll S, Bolte G, Brand T, et al. How to tackle key challenges in the promotion of physical activity among older adults (65+): the AEQUIPA network approach. International journal of environmental research and public health. 2017;14(4):379.
20. Greaney ML, Lees FD, Blissmer BJ, Riebe D, Clark PG. Psychosocial Factors Associated With Physical Activity in Older Adults. Annual Review of Gerontology & Geriatrics. 2016;36:273.
21. Baert V, Gorus E, Mets T, Geerts C, Bautmans I. Motivators and barriers for physical activity in the oldest old: A systematic review. Ageing Research Reviews. 2011;10(4):464-74.

22. Warner LM, Wolff JK, Spuling SM, Wurm S. Perceived somatic and affective barriers for self-efficacy and physical activity. Journal of health psychology. 2017:1359105317705979.
23. Rhodes RE, Martin AD, Taunton JE, Rhodes EC, Donnelly M, Elliot J. Factors associated with exercise adherence among older adults. An individual perspective. Sports Medicine. 1999;28(6):397-411.
24. Direito A, Walsh D, Hinbarji M, Albatal R, Tooley M, Whittaker R, et al. Using the Intervention Mapping and Behavioral Intervention Technology Frameworks: Development of an mHealth Intervention for Physical Activity and Sedentary Behavior Change. Health Education & Behavior. 2017:1090198117742438.
25. Martin KA, Sinden AR. Who will stay and who will go? A review of older adults' adherence to randomized controlled trials of exercise. Journal of Aging and Physical Activity. 2001;9(2):91-114.
26. Van Dyck D, Cardon G, De Bourdeaudhuij I. Which psychological, social and physical environmental characteristics predict changes in physical activity and sedentary behaviors during early retirement? A longitudinal study. PeerJ. 2017;5:e3242.
27. Smith GL, Banting L, Eime R, O'Sullivan G, van Uffelen JG. The association between social support and physical activity in older adults: a systematic review. International Journal of Behavioral Nutrition and Physical Activity. 2017;14(1):56.
28. Clarke CL, Sniehotta FF, Vadiveloo T, Argo IS, Donnan PT, McMurdo ME, et al. Factors associated with change in objectively measured physical activity in older people–data from the physical activity cohort Scotland study. BMC geriatrics. 2017;17(1):180.
29. Manz K, Mensink GB, Jordan S, Schienkiewitz A, Krug S, Finger JD. Predictors of physical activity among older adults in Germany: a nationwide cohort study. BMJ open. 2018;8(5):e021940.
30. Notthoff N, Reisch P, Gerstorf D. Individual Characteristics and Physical Activity in Older Adults: A Systematic Review. Gerontology. 2017;63(5):443-59.
31. Devereux-Fitzgerald A, Powell R, Dewhurst A, French DP. The acceptability of physical activity interventions to older adults: A systematic review and meta-synthesis. Social Science & Medicine. 2016;158:14-23.
32. Schutzer KA, Graves BS. Barriers and motivations to exercise in older adults. Prev Med. 2004;39(5):1056-61.
33. Normansell R, Smith J, Victor C, Cook DG, Kerry S, Iliffe S, et al. Numbers are not the whole story: a qualitative exploration of barriers and facilitators to increased physical activity in a primary care based walking intervention. BMC Public Health. 2014;14(1):1272.
34. Tang MY, Smith DM, Mc Sharry J, Hann M, French DP. Behavior Change Techniques Associated With Changes in Postintervention and Maintained Changes in Self-Efficacy For Physical Activity: A Systematic Review With Meta-analysis. Annals of Behavioral Medicine. 2018;53(9):801-15.
35. Notthoff N, Klomp P, Doerwald F, Scheibe S. Positive messages enhance older adults' motivation and recognition memory for physical activity programmes. European journal of ageing. 2016;13(3):251-7.
36. Peels DA, van Stralen MM, Bolman C, Golsteijn RHJ, de Vries H, Mudde AN, et al. The differentiated effectiveness of a printed versus a Web-based tailored physical activity intervention among adults aged over 50. Health Education Research. 2014;29(5):870-82.
37. Lara J, O'Brien N, Godfrey A, Heaven B, Evans EH, Lloyd S, et al. Pilot Randomised Controlled Trial of a Web-Based Intervention to Promote Healthy Eating, Physical Activity and Meaningful Social Connections Compared with Usual Care Control in People of Retirement Age Recruited from Workplaces. PLOS ONE. 2016;11(7):e0159703.

38. Peels DA, de Vries H, Bolman C, Golsteijn RHJ, van Stralen MM, Mudde AN, et al. Differences in the use and appreciation of a web-based or printed computer-tailored physical activity intervention for people aged over 50 years. Health Education Research. 2013;28(4):715-31.
39. Peels DA, Bolman C, Golsteijn RHJ, de Vries H, Mudde AN, van Stralen MM, et al. Long-term efficacy of a printed or a Web-based tailored physical activity intervention among older adults. International Journal of Behavioral Nutrition and Physical Activity. 2013;10(1):104.
40. Gardner B, Smith L, Lorencatto F, Hamer M, Biddle SJH. How to reduce sitting time? A review of behaviour change strategies used in sedentary behaviour reduction interventions among adults. Health Psychology Review. 2016;10(1):89-112.
41. Chastin SFM, Fitzpatrick N, Andrews M, DiCroce N. Determinants of sedentary behavior, motivation, barriers and strategies to reduce sitting time in older women: a qualitative investigation. Int J Environ Res Public Health. 2014;11(1):773-91.
42. Chastin SFM, Buck C, Freiberger E, Murphy M, Brug J, Cardon G, et al. Systematic literature review of determinants of sedentary behaviour in older adults: a DEDIPAC study. International Journal of Behavioral Nutrition and Physical Activity. 2015;12(1):127.
43. Owen N, Sugiyama T, Eakin EE, Gardiner PA, Tremblay MS, Sallis JF. Adults' Sedentary Behavior: Determinants and Interventions. American Journal of Preventive Medicine. 2011;41(2):189-96.
44. Brug J, Chinapaw M. Determinants of engaging in sedentary behavior across the lifespan; lessons learned from two systematic reviews conducted within DEDIPAC. International Journal of Behavioral Nutrition and Physical Activity. 2015;12(1):134.
45. Fiatarone Singh MA, Gates N, Saigal N, Wilson GC, Meiklejohn J, Brodaty H, et al. The Study of Mental and Resistance Training (SMART) study-resistance training and/or cognitive training in mild cognitive impairment: a randomized, double-blind, double-sham controlled trial. J Am Med Dir Assoc. 2014;15(12):873-80.
46. Clemson L, Fiatarone Singh MA, Bundy A, Cumming RG, Manollaras K, O'Loughlin P, et al. Integration of balance and strength training into daily life activity to reduce rate of falls in older people (the LiFE study): randomised parallel trial. BMJ : British Medical Journal. 2012;345:e4547.
47. Clemson L, Munro J, Singh MF. Lifestyle-integrated Functional Exercise (LiFE) program to prevent falls: trainer's manual: Sydney University Press; 2014.
48. Haesner M, Steinert A, O'Sullivan JL, Weichenberger M. Evaluating an online cognitive training platform for older adults: User experience and implementation requirements. Journal of gerontological nursing. 2015;41(8):22-31.
49. Gates NJ, Sachdev PS, Fiatarone Singh MA, Valenzuela M. Cognitive and memory training in adults at risk of dementia: A Systematic Review. BMC Geriatrics. 2011;11(1):55.
50. Corbett A, Owen A, Hampshire A, Grahn J, Stenton R, Dajani S, et al. The effect of an online cognitive training package in healthy older adults: an online randomized controlled trial. Journal of the American Medical Directors Association. 2015;16(11):990-7.
51. Kueider AM, Parisi JM, Gross AL, Rebok GW. Computerized Cognitive Training with Older Adults: A Systematic Review. PLOS ONE. 2012;7(7):e40588.
52. Willis SL, Tennstedt SL, Marsiske M, Ball K, Elias J, Koepke KM, et al. Long-term effects of cognitive training on everyday functional outcomes in older adults. Jama. 2006;296(23):2805-14.
53. McDougall Jr GJ. Cognitive interventions among older adults. Annual review of nursing research. 1999;17:219-40.
54. Pike KE, Chong MS, Hume CH, Keech BJ, Konjarski M, Landolt KA, et al. Providing Online Memory Interventions for Older Adults: A Critical Review and Recommendations for Development. Australian Psychologist. 2018.

55. Mohan J. Computerized cognitive training for the elderly: A study evaluating use of the Brain Trainer Plus™ in care homes for elderly people 2015.
56. Haesner M, O'Sullivan JL, Gövercin M, Steinhagen-Thiessen E. Requirements of older adults for a daily use of an internet-based cognitive training platform. Informatics for Health and Social Care. 2015;40(2):139-53.
57. Valls-Pedret C, Sala-Vila A, Serra-Mir M, Corella D, de la Torre R, Martínez-González MÁ, et al. Mediterranean diet and age-related cognitive decline: a randomized clinical trial. JAMA internal medicine. 2015;175(7):1094-103.
58. Petroka K, Campbell-Bussiere R, Dychtwald DK, Milliron B-J. Barriers and facilitators to healthy eating and disease self-management among older adults residing in subsidized housing. Nutrition and Health. 2017;23(3):167-75.
59. Seguin R, Connor L, Nelson M, LaCroix A, Eldridge G. Understanding Barriers and Facilitators to Healthy Eating and Active Living in Rural Communities. Journal of Nutrition and Metabolism. 2014;2014:8.
60. Kelly S, Martin S, Kuhn I, Cowan A, Brayne C, Lafortune L. Barriers and Facilitators to the Uptake and Maintenance of Healthy Behaviours by People at Mid-Life: A Rapid Systematic Review. PLoS One. 2016;11(1):e0145074.
61. Nicklett EJ, Kadell AR. Fruit and vegetable intake among older adults: a scoping review. Maturitas. 2013;75(4):305-12.
62. Lo YT, Chang YH, Lee MS, Wahlqvist ML. Health and nutrition economics: diet costs are associated with diet quality. Asia Pacific journal of clinical nutrition. 2009;18(4):598-604.
63. Payette H, Shatenstein B. Determinants of healthy eating in community-dwelling elderly people. Canadian Journal of Public Health. 2005;96(SUPPL. 3):S27-S31.
64. Bloom I, Edwards M, Jameson KA, Syddall HE, Dennison E, Gale CR, et al. Influences on diet quality in older age: the importance of social factors. Age and ageing. 2017;46(2):277-83.
65. Bloom I, Lawrence W, Barker M, Baird J, Dennison E, Sayer AA, et al. What influences diet quality in older people? A qualitative study among community-dwelling older adults from the Hertfordshire Cohort Study, UK. Public health nutrition. 2017;20(15):2685-93.
66. Robinson SM. Improving nutrition to support healthy ageing: what are the opportunities for intervention? Proceedings of the Nutrition Society. 2017:1-8.
67. Appleton KM, Dinnella C, Spinelli S, Morizet D, Saulais L, Hemingway A, et al. Consumption of a High Quantity and a Wide Variety of Vegetables Are Predicted by Different Food Choice Motives in Older Adults from France, Italy and the UK. Nutrients. 2017;9(9):923.
68. Sahyoun NR, Zhang XL, Serdula MK. Barriers to the consumption of fruits and vegetables among older adults. Journal of Nutrition for the Elderly. 2006;24(4):5-21.
69. Greene GW, Lofgren I, Paulin C, Greaney ML, Clark PG. Differences in psychosocial and behavioral variables by dietary screening tool risk category in older adults. Journal of the Academy of Nutrition and Dietetics. 2018;118(1):110-7.
70. O'Brien N, McDonald S, Araújo-Soares V, Lara J, Errington L, Godfrey A, et al. The features of interventions associated with long-term effectiveness of physical activity interventions in adults aged 55–70 years: a systematic review and meta-analysis. Health Psychology Review. 2015;9(4):417-33.
71. Stiggelbout M, Hopman-Rock M, Crone M, Lechner L, van Mechelen W. Predicting older adults' maintenance in exercise participation using an integrated social psychological model. Health Educ Res. 2006;21(1):1-14.

72. Brooker H, Wesnes KA, Ballard C, Hampshire A, Aarsland D, Khan Z, et al. An online investigation of the relationship between the frequency of word puzzle use and cognitive function in a large sample of older adults. International Journal of Geriatric Psychiatry. 2019;34(7):921-31.
73. Rolstad S, Adler J, Rydén A. Response Burden and Questionnaire Length: Is Shorter Better? A Review and Meta-analysis. Value in Health. 2011;14(8):1101-8.
74. Iglesias C, Torgerson D. Does length of questionnaire matter? A randomised trial of response rates to a mailed questionnaire. Journal of health services research & policy. 2000;5(4):219-21.
