# Supplementary figures and images for: Planning and optimising a digital intervention to protect older adults’ cognitive health
Source: Pilot Feasibility Stud. 2021 Aug 18;7:158. doi: 10.1186/s40814-021-00884-2 (PMC8371874; doi:10.1186/s40814-021-00884-2)

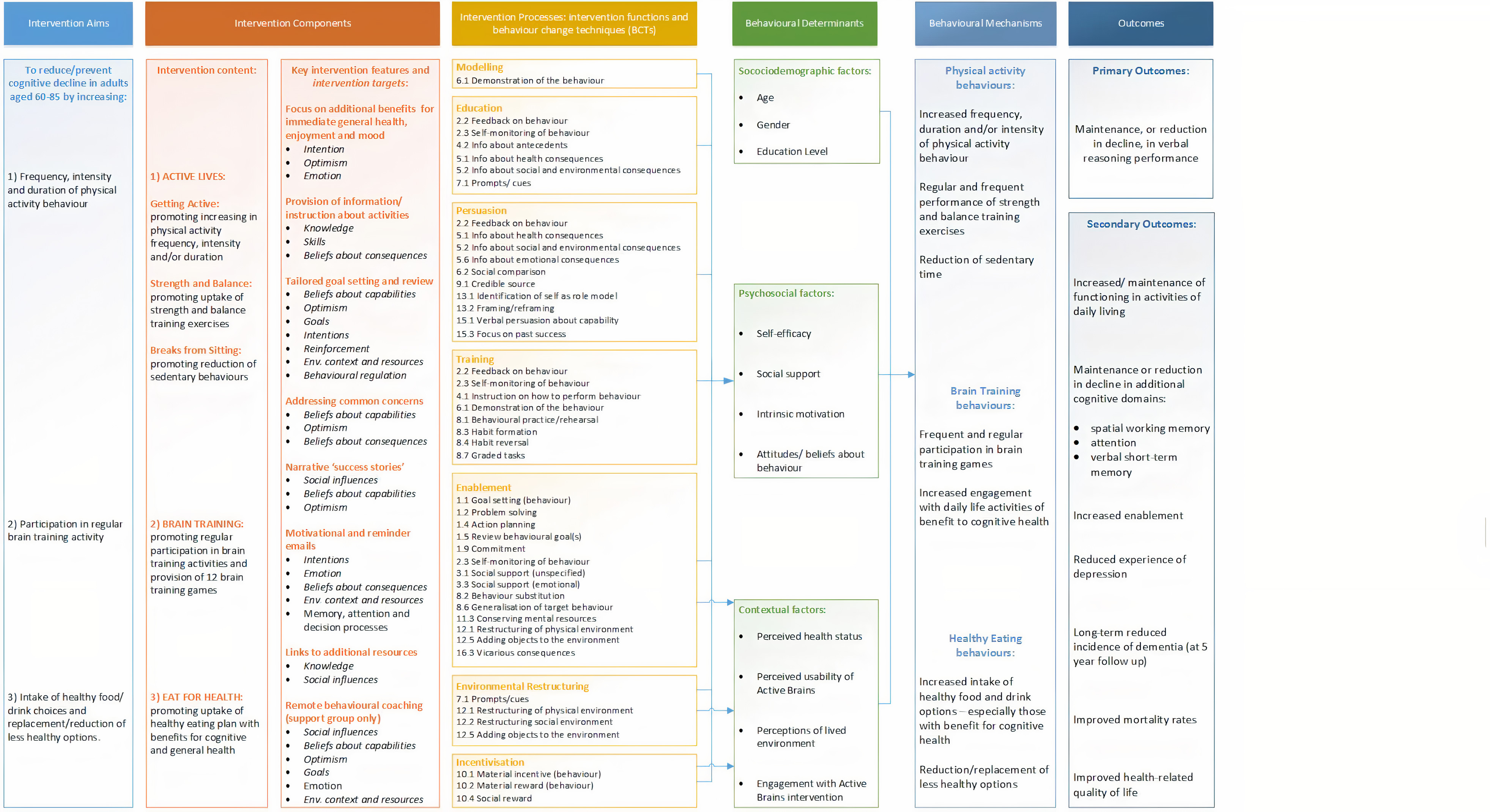

Supplement: Supplementary file 5 — Additional file 5:. Additional Figure 1 (.jpg) - Full Active Brains intervention logic model [file 40814_2021_884_MOESM5_ESM.jpg]
